# A key to species of subgenus *Lithochlaenius* (Coleoptera, Carabidae, Chlaeniini, *Chlaenius*), with descriptions of three new species

**DOI:** 10.3897/zookeys.128.1804

**Published:** 2011-09-09

**Authors:** Ye Valim, David H. Kavanaugh, Hongliang Shi, Hongbin Liang

**Affiliations:** 1 Key Laboratory of Zoological Systematics and Evolution, Institute of Zoology, Chinese Academy of Sciences, Beijing 100101, China; 2 Key Laboratory for Plant Pests Management of Mountainous Region, Institute of Entomology, Guizhou, University, Guiyang 550025, China; 3 Department of Entomology, California Academy of Science, San Francisco, California 94118, U.S.A.

**Keywords:** Coleoptera, Carabidae, *Chlaenius*, *Lithochlaenius*, new species, new synonymy, key

## Abstract

Three new species of genus *Chlaenius* Bonelli subgenus *Lithochlaenius* Kryzhanovskij are described from China: *Chlaenius chuanqianensis* Liu & Liang, **sp. n.** (type locality: Xishui, Guizhou Province), *Chlaenius linwensini* Liu & Liang, **sp. n.** (type locality: Fujian Province), and *Chlaenius propeagilis* Liu & Kavanaugh, **sp. n.** (type locality: Gaoligongshan, Yunnan Province). Seven species of the subgenus are redescribed: *Chlaenius agiloides* Jedlička, *Chlaenius formosensis* Lorenz, *Chlaenius agilis* Chaudoir, *Chlaenius leishanensis* Kirschenhofer, *Chlaenius noguchii* Bates, *Chlaenius rambouseki* Lutshnik, and *Chlaenius wrasei* Kirschenhofer. Additional taxonomic changes include the following: *Chlaenius formosanus* Jedlička is treated as a junior synonym of *Chlaenius rambouseki* Lutshnik and *Chlaenius anchomenoides* Bates, **syn. n.** and *Chlaenius nuristanus* Jedlička as junior synonyms of *Chlaenius agilis* Chaudoir, **syn. n.** *Chlaenius latro*LaFerté-Sénectère is considered a *nomen nudum* stat. n. and unavailable, leaving *Chlaenius agilis*Chaudoir as the next available name. *Chlaenius nuristanus*aberration *rubridipes*Jedlička is also an unavailable name. *Chlaenius formosensis*Lorenz (=*Chlaenius formosanus*Habu) is returned to species status stat. n. A key to adults of the 10 known species of subgenus *Lithochlaenius* is provided.

## Introduction

*Lithochlaenius* Kryzhanovskij (1976) is one of the subgenera included in genus *Chlaenius* Bonelli (1810) of the carabid tribe Chlaeniini. This subgenus was erected for adults with cordate pronota and long, narrow metepisterna. Members of this subgenus are very similar to those of subgenus *Stenochlaenius*, with which they share a cordate pronotum, but from which they differ in having a pubescent body (body glabrous in *Stenochlaenius*adults).

To date, nine species of the subgenus have been described from Asia, namely, *Chlaenius agiloides* Jedlička (1935) (Type locality: Wenxian, Gansu, China), *Chlaenius agilis* Chaudoir (1856) (Nord Industan, India), *Chlaenius anchomenoides* Bates (1889) (Goorais Valley, Pakistan), *Chlaenius formosanus* Jedlička (1935) (Kosempo, Taiwan, China), *Chlaenius formosensis* Lorenz (1998) (Urai, Taiwan, China), *Chlaenius leishanensis* Kirschenhofer (2005) (Leishan, Guizhou, China), *Chlaenius noguchii* Bates (1873) (Kawachi, Japan), *Chlaenius rambouseki* Lutshnik (1933) (Ussuri, Far East, Russia), and *Chlaenius wrasei* Kirschenhofer 1997 (Wenxian, Gansu, China). Recently, after studying *Chlaenius* specimens in the collection of the National Zoological Museum of China (Beijing) and in several other museums, we determined that two of these, *Chlaenius anchomenoides* Bates and *Chlaenius formosanus* Jedlička, were just junior synonyms of other species names, and that some specimens collected from Guizhou, Sichuan, Yunnan, and Fujian provinces represented three new species. In this presentation, the new synonymic relationships are formally proposed and descriptions of the three new species are provided.

To date, no key including all known species of *Lithochlaenius* has been published. Based on our study of type specimens and/or original descriptions, we provide here a key to aid identifications of adults of all known species, including the three new ones described in this paper.

## Materials and methods

We measured all available specimens for each species except for those with plentiful specimens, for which five males and five females were measured, including smallest and largest specimens for each sex (determined by visual inspection of the assembled samples). Measurements were made with the aid of a Nikon SMZ1500 stereoscopic dissecting microscope with a micrometer. Body length (BL) was measured as the linear distance along the midline from the apex of the longer mandible to the apex of the longer elytron. Other measurements, and abbreviations used for them in this paper, are as follows: HW = maximum head width including the eyes; EYL = eye length measured along the longitudinal diameter of the eye (dorsal–lateral view); PL = length of pronotum measured along median line; PW = pronotum width at its widest point; EL = elytron length from base to apex; EW = width across both elytra at widest point (equal to body width).

Wherever we refer to abdominal ventral plates, we use the numbering system that recognizes the generally accepted segmental homologies in Carabidae. Thus the first visible sternum (i.e. the sternum divided medially by the hind coxae) in *Chlaenius* adults is sternum II and the last visible sternum is sternum VII.

All photographs were taken through a Nikon stereoscopic dissecting microscope fitted with a Canon 450D camera, and were edited by Helicon Focus and Photoshop software.

Specimens examined in the course of this study were deposited at the following collections:

BMNH	Natural History Museum, London, U.K.

CASC	California Academy of Sciences, San Francisco, U.S.A.

CCCC	Private Collection of Changchin Chen, Tianjin, China

HBUM	Museum of Hebei University, Baoding, China

IZCAS	National Zoological Museum of China, Institute of Zoology, Beijing, China

MNHN	Museum National d'Histoire Naturelle, Paris, France

NMPC	Narodni Muzeum, Prirodovedecke Muzeum, Prague, Czech Republic

OMNH	Osaka Museum of Natural History, Osaka, Japan

SIECAS	Institute of Plant Physiology & Ecology, Shanghai Institutes for Biological Sciences, Chinese Academy of Sciences, Shanghai, China

ZRAS	Zoological Institute, Russian Academy of Sciences, St. Petersburg, Russia

## Taxonomy

### Subenus Lithochlaenius

Kryzhanovskij, 1976

http://species-id.net/wiki/Lithochlaenius

Hemichlaenius  Lutshnik, 1933:169 (nec Bates, 1892:307). Type species *Chlaenius rambouseki* Lutshnik, 1933; Kryzhanovskij, 1976:11
Lithochlaenius  Kryzhanovskij, 1976:9. Type species *Chlaenius rambouseki* Lutshnik, 1933; Morita, 1993:161
Agilochlaenius  Kirschenhofer, 1997:116. Type species *Chlaenius latro* LaFerté–Sénectère, 1851; Kirschenhofer, 2000:58

#### Diagnosis.

Antennomere 3 distinctly longer than antennomeres 1 and 2 combined (Fig. 9); pronotum distinctly cordate (Fig. 1), disk glabrous or sparsely pubescent, each hind angle with single seta (Fig. 2); scutellar setiferous pore puncture present (Fig. 3); venter densely pubescent laterally, sparsely pubescent or glabrous medially (Fig. 4); prosternal intercoxal process punctate, bordered at apex; metepisterna long and narrow (Fig. 10); basal margination of elytra incomplete, absent from medial portion; anterior tarsomere 4 short and deeply emarginate apically (more distinctly so in male, Figs 5–8); aedeagus tubular, ejaculatory orifice long, extended to basal fifth of aedeagus, lamella short (Figs 11, 117–145); gonostylus of female ovipositor smooth, with one setiferous pore near apex and one slender spine at basal inner margin (Figs 12–15).

#### Description.

 Length 12.0–18.0 mm, width 4.6–6.4 mm. Head and pronotum black, with green, blue or coppery metallic luster; elytra black, with blue or coppery luster in a few species; ventral surface black; legs black, brown or yellow; antennae yellow, brown, or dark brown.

Head with vertex nearly glabrous, or sparsely and coarsely punctate behind posterior level of eye; eyes moderately prominent; genae pubescent; antennae long, antennomere 1 (scape) coniform (Figs 52–53, 57–59), cylindrical (Figs 56, 60), or elongate-ovoid (Figs 54–55, 61–65); antennomere 3 sparsely setose, distinctly longer than antennomeres 1 and 2 combined (Fig. 9); mandibles triangular, hooked at apex; labrum with six setigerous punctures near apex; maxillary and labial palpi cylindrical, slightly compressed at apex; maxillary palpi glabrous; penultimate labial palpomere with a few setae, apical labial palpomere glabrous; glossal sclerite with two subapical setae; tooth of mentum bifid or emarginate at apex, with one pair of setae near the base; gula glabrous, slightly rugose.

**Figures 1–15.**
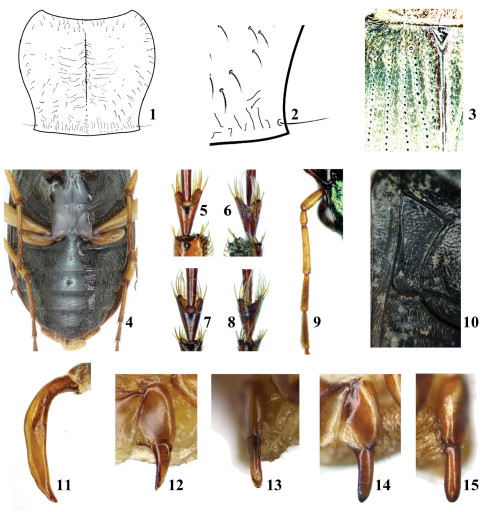
Character states of *Chlaenius (Lithochlaenius)* spp. **Figs 1–13** *Chlaenius rambouseki* Lutshnik**1** pronotum**2** site of hind angle seta**3** elytral base showing the site of basal pore**4** abdominal sterna showing the pubescence**5** male anterior tarsomere 4 in dorsal view**6** male anterior tarsomere 4 in ventral view**7** female anterior tarsomere 4 in dorsal view**8** female anterior tarsomere 4 in ventral view**9** antennomeres 1–5 showing antennomere 3 distinctly longer than 1 and 2 ones combined**10** metepisternum**11** aedeagus, showing the basic structure of the aedeagus in *Lithochlaenius* species**12** female gonostylus in ventral view**13** female gonostylus in lateral view**14** female gonostylus of *Chlaenius propeagilis* sp. n. in ventral view**15** female gonostylus of *Chlaenius propeagilis* sp. n. in lateral view.

Pronotum cordate (Fig. 1), widest at apical one-third, front angles obtuse, hind angles acute; lateral margins bordered; disk glabrous or sparsely pubescent, base longitudinally rugose, punctate; basal foveae small, deep, rugose, pubescent; hind angles each with one seta (Fig. 2).

Elytra moderately convex, oblong, with distinct isodiametric microsculpture at least laterally; scutellar striae long, with basal setiferous pore present (Fig. 3); at least outer intervals pubescent (Figs 80–93), pubescence on intervals 8 and 9 generally denser than on others; wings full-sized, functional.

Abdominal sterna densely pubescent laterally, sparsely pubescent or glabrous medially (Fig. 4); sterna IV to VI with single long seta at each side; sternum VII with one seta at each side in male, two in female; apex of sternum VII more rounded in male than in female.

Pro- and mesosterna and pro-, mes-, and metepisterna densely pubescent; metasternum pubescent laterally, nearly glabrous medially; prosternal intercoxal process punctate and bordered at apex; scutellum triangular, glabrous.

Anterior femora without tooth; tarsomeres short, sparsely setose dorsally; anterior tarsomere 4 short and triangular, deeply emarginate (more so in male), with two rows of long setae ventrally (Figs 5–8); basal three anterior tarsomeres dilated in male.

Male genitalia with aedeagus tubular, simple; ejaculatory orifice long (Fig. 11); apical lamella short, rounded or slightly truncated at apex. Gonostylus of female ovipositor smooth, with one setiferous pore near apex and a long slender spine at basal inner margin, outer margin without or with a very short spine (Figs 12–15).

**Figures 16–21.**
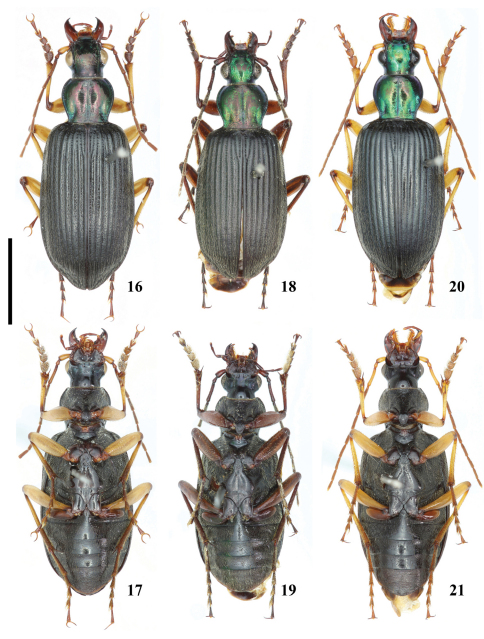
Habitus of *Chlaenius (Lithochlaenius)* spp**16** *Chlaenius chuanqianensis* sp. n., holotype, male, dorsal view**17** *Chlaenius chuanqianensis* sp. n., holotype, male, ventral view**18** *Chlaenius linwensini* sp. n., holotype, male, dorsal view**19** *Chlaenius linwensini* sp.n., holotype, male, ventral view**20** *Chlaenius propeagilis* sp. n., holotype, male, dorsal view**21** *Chlaenius propeagilis* sp. n., holotype, male, ventral view. Scale line = 5.0 mm.

**Figures 22–27.**
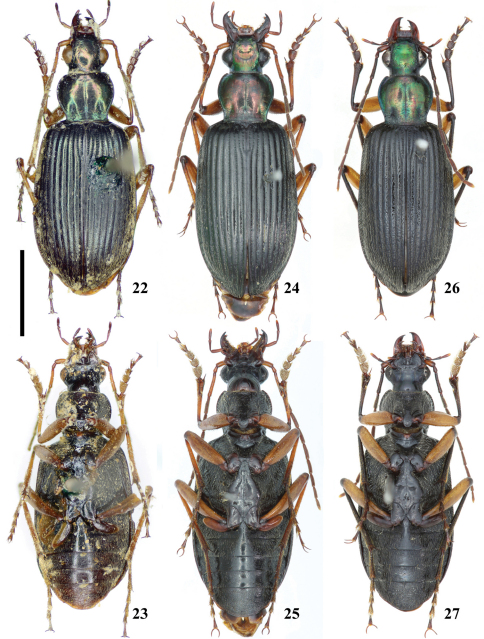
Habitus of*Chlaenius (Lithochlaenius)* spp**22** *Chlaenius agilis* Chaudoir, holotype, dorsal view**23** *Chlaenius agilis* Chaudoir, holotype, ventral view**24** *Chlaenius formosensis* Lorenz, male, in CCCC, dorsal view**25** *Chlaenius formosensis* Lorenz, ventral view**26** *Chlaenius leishanensis* Kirschenhofer, male, in IZCAS, dorsal view**27** *Chlaenius leishanensis* Kirschenhofer, male, in IZCAS, ventral view. Scale line = 5.0 mm.

**Figures 28–33.**
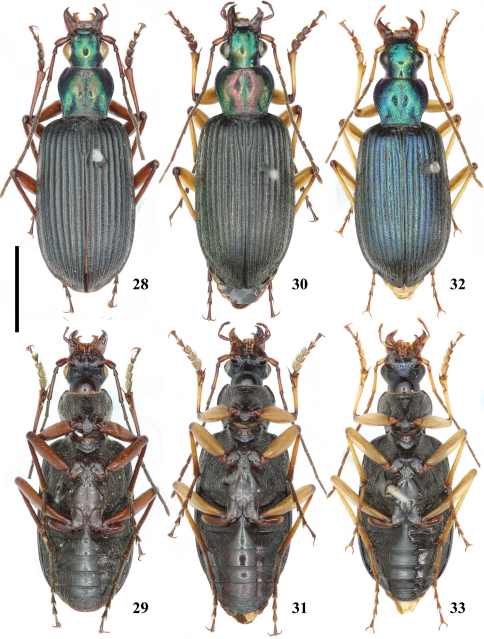
*Chlaenius (Lithochlaenius)* spp**28** *Chlaenius noguchii* Bates, male, in IZCAS, dorsal view**29** *Chlaenius noguchii* Bates, male, in IZCAS, ventral view**30***Chlaenius wrasei* Kirschenhofer, male, in IZCAS, dorsal view**31** *Chlaenius wrasei* Kirschenhofer, male, in IZCAS, ventral view**32** *Chlaenius agiloides* Jedlička, male, in IZCAS, dorsal view**33** *Chlaenius agiloides* Jedlička, male, in IZCAS, ventral view. Scale line = 5.0 mm.

**Figures 34–39.**
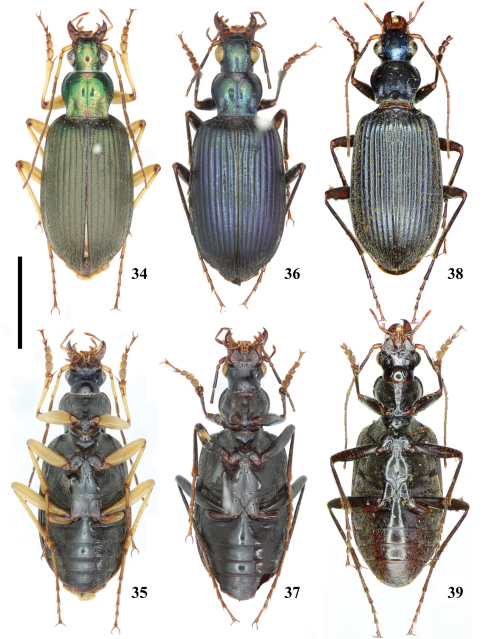
*Chlaenius (Lithochlaenius)* spp**34** *Chlaenius rambouseki* Lutshnik, male, collected from Ussuri region, in IZCAS, dorsal view**35** *Chlaenius rambouseki* Lutshnik, male, collected from Ussuri region, in IZCAS, ventral view**36** *Chlaenius anchomenoides* Bates, cotype, male, in BMNH, dorsal view**37** *Chlaenius anchomenoides* Bates, cotype, male, in BMNH, ventral view**38** *Chlaenius nuristanus* Jedlička, paratype, male, in MNHN, dorsal view**39** *Chlaenius nuristanus* Jedlička, paratype, male, in MNHN, ventral view. Scale line = 5.0 mm.

**Figures 40–45.**
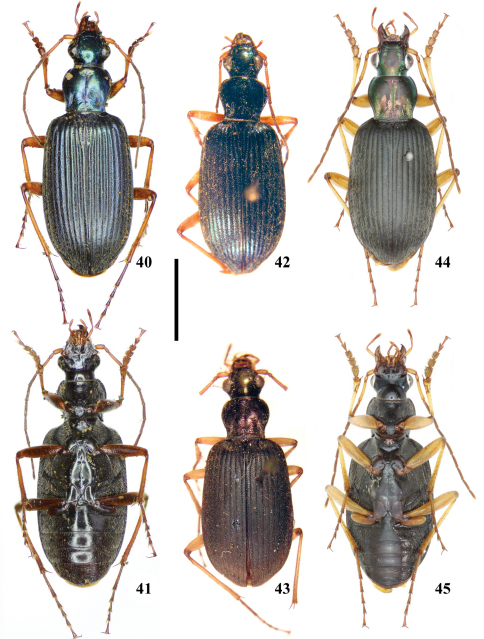
*Chlaenius (Lithochlaenius)* spp**40** *Chlaenius nuristanus* a. *rubridipes* Jedlička, paratype, male, in MNHN, dorsal view**41** *Chlaenius nuristanus* a. *rubridipes* Jedlička, paratype, male, in MNHN, ventral view**42** *Chlaenius agiloides* Jedlička, holotype, male, in NMPC, dorsal view**43***Chlaenius formosanus* Jedlička, holotype, male, in NMPC, dorsal view**44** *Chlaenius rambouseki* Lutshnik, from Taiwan, male, in IZCAS, dorsal view**45** *Chlaenius rambouseki* Lutshnik, from Taiwan, male, in IZCAS, ventral view. Scale line = 5.0 mm.

#### Geographical Distribution.

 China (Heilongjiang, Liaoning, Shaanxi, Gansu, Henan, Hubei, Hunan, Zhejiang, Fujian, Taiwan, Jiangxi, Guangdong, Hainan, Guangxi, Guizhou, Sichuan, Yunnan, Tibet), North Korea, Japan, Russia (Far East), Afghanistan, Pakistan, India. The known localities of *Lithochlaenius* species are shown in Fig. 158. Based on the work of Andrewes (1930) and Paik et al. (2006), members of this subgenus also occur in Indo-China (Laos, Cambodia, Vietnam), but we have not studied specimens from those countries.

#### Biology.

Members of this subgenus are typically collected on sandy beaches of rivers or streams (Figs 146–153). Adults of some species (e. g., *Chlaenius agiloides*, *Chlaenius rambouseki*) have been observed feeding on mollusks, worms, and dragonfly larvae at night (Figs 154–157). A few species have also been collected in light traps.

#### Remarks.

 Based on the metallic body surface, single supraorbital seta, antennomere 3 longest and antennomeres 4–11 densely pubescent, *Lithochlaenius* species can be recognized as a member of the genus *Chlaenius*. Members of this subgenus are similar to those of subgenus *Stenochlaenius* in shape of the pronotum, but the latter are much smaller and have a glabrous body surface.

As presently conceived, the subgenus can be divided into two species groups: 1) the *rambouseki* group, members of which have all elytral intervals densely and more or less equally pubescent, and males have aedeagi slender in dorsal view; and 2) the *agilis* group, members of which have at least elytral intervals 1–5 glabrous medially with pubescence restricted to the strial depressions, intervals 6–9 densely pubescent, and males have aedeagi generally stouter in dorsal view. Most species of the subgenus have restricted geographical ranges, and only C. *rambouseki*is more broadly distributed.

In his treatment of the North American species of genus *Chlaenius*, Bell (1960) suggested that his *solitarius* species group, which included *Chlaenius cordicollis* Kirby, *Chlaenius leucoscelis* Chevrolat, *Chlaenius prasinus* Dejean, *Chlaenius purpureus* Chaudoir, and *Chlaenius solitarius* Say, represented a distinct group within his subgenus *Chlaenius* sensu stricto. He noted that the range of this group extends south into South America and that "Related forms occur in the Old World”. In the paper in which he proposed *Lithochlaenius* as a replacement name for *Hemichlaenius* Lutshnik, Kryzhanovskij (1976: 16) cited Bell's paper and suggested that *Chlaenius solitarius* might be a North American representative of that subgenus. Robert Davidson (personal communication) shares the view that species of Bell's *solitarius* group, and at least five additional species in Middle and South America, are likely related to some if not all *Lithochlaenius* species. All of these New World species share most of the diagnostic features of *Lithochlaenius*and all of them have elytral pubescence as seen in members of the *rambouseki* group. However, members of all these New World species are distinguished in having the lateral and basal elytral margins smoothly continuous around the humeri without forming any trace of an angle and the elytral epipleura and basal regions smoothly continuous around the humeri, not separated by a carina of any kind. These were the main features that Bell used to distinguish members of his *solitarius* group. In contrast, members of all the Asian species of *Lithochlaenius* that we have studied have a distinct humeral angle formed at the junction of the lateral and basal elytral margins and a more or less distinctly carinate separation of the (lateral) epipleural from the basal (anterior vertical) elytral surfaces. Hence, Asian *Lithochlaenius* specimens could not be identified as members of the *solitarius*groups using Bell's (1960) key. There is also greater variation in the development of the elytral basal margin (from complete to partially interrupted) and in the length and shape of the apical lamella of the male aedeagus among New World species than we have seen among the Asian *Lithochlaenius*species. Consequently, we suggest that placement of any New World species in subgenus *Lithochlaenius* would be premature at this time and should await a more comprehensive, worldwide treatment of genus *Chlaenius* and analyses of phylogenetic relationships among the included species, species groups, and subgenera.

#### Key to the species of subgenus Lithochlaenius

**Table:** 

1	All intervals punctate, pubescent, slightly convex (Figs 88–89, 93); aedeagus slender, basal portion rugose (*rambouseki* group)	**2**
–	At least basal half of intervals 1–5 smooth and glabrous medially, distinctively convex (Figs 80–87, 90–92); aedeagus usually stout, basal portion smooth (*agilis* group)	3
2	Intervals dull, with dense and regular punctures (Figs 89, 93); antennomere 1 elongate ovoid (Figs 61, 65); apical lamella of aedeagus thin (Figs 137, 145)	*Chlaenius rambouseki* Lutshnik
–	Intervals shining, with sparse and irregular punctures (large and small punctures mixed) (Fig. 88); antennomere 1 cylindrical (Fig. 60); apical lamella of aedeagus thick (Figs 134–135)	*Chlaenius agiloides* Jedlička
3	Antennomere 1 with apical end much thicker than basal end, coniform (Figs 52–53, 57–59)	4
–	Antennomere 1 with apical end as thick as basal end, elongate-ovoid (Figs 54, 55, 62–64) or cylindrical (Fig. 56)	8
4	Intervals 1–7 glabrous medially, with a row of pubescence laterally (near striae) (Fig. 86); legs dark brown or brown (Fig. 29); apex of sternum VII subtruncate (Figs 105, 106); aedeagus slender, depressed, apical lamella bent ventrally (Figs 129–130)	*Chlaenius noguchii* Bates
–	Intervals 6–7 pubescent, intervals 1–5 glabrous medially, with a row of pubescence laterally (near striae) (Figs 80–81, 85, 87); aedeagus stout	5
5	Tibiae and femora bi-colored, with tibiae black or dark brown (Figs 26–27), femora yellow or light brown; lamella of aedeagus rounded at apex (Fig. 127), thickened, and bent ventrally (Fig. 128)	*Chlaenius leishanensis* Kirschenhofer
–	Tibiae and femora concolorous, yellow or brown (Figs 16–19, 30–31)	6
6	All antennomeres brown or dark brown (Fig. 18); hind trochanters brown, nearly the same color as hind femora (Fig. 19); elytra with basal three-fourths of intervals 1–5 glabrous medially; lamella of aedeagus sub-truncate at apex (Fig. 119), thick (Fig. 120)	*Chlaenius linwensini* Liu & Liang, sp. n.
–	At least antennomere 1 yellow or brown, paler than the rest (Figs 16, 30); hind trochanters much darker than hind femora (Figs 17, 41); elytra with entire lengths of intervals 1–5 glabrous medially; lamella of aedeagus rounded at apex (Figs 117, 131), thin (Figs 118, 132)	7
7	Antennomeres 1–3 brown, color paler than antennomeres 4–11 (Fig. 30); apical lamella of aedeagus bent ventrally (Fig. 132)	*Chlaenius wrasei* Kirschenhofer
–	Only antennomere 1 yellow or brown, color paler than antennomeres 2–11 (Fig. 16); apical lamella of aedeagus straight (Fig. 118)	*Chlaenius chuanqianensis* Liu & Liang, sp. n.
8	Intervals 1–7 glabrous medially (Fig. 84); antennomere 1 cylindrical (Fig. 56); lamella of aedeagus subtruncate at apex (Fig. 125)	*Chlaenius formosensis* Lorenz
–	At most intervals 1–5 glabrous medially (Figs 82–83, 90–92); antennomere 1 elongate ovoid (Figs 54–55, 62–64); lamella of aedeagus round at apex (Figs 121, 123, 138, 140, 142)	9
9	Male with media lobe of aedeagus gradually bent near base (Fig. 122), lamella triangular at apex, left side of media lobe nearly straight in dorsal view (Fig. 121)	*Chlaenius propeagilis* Liu & Kavanaugh, sp. n.
–	Male with media lobe of aedeagus abruptly bent near the base (Figs 124, 139, 141, 143), lamella rounded at apex, left side of media lobe expand laterally in dorsal view (Figs 123, 138, 140, 142)	*Chlaenius agilis* Chaudoir

##### Theagilisgroup

### Chlaenius Lithochlaenius chuanqianensis

Liu & Liang sp. n.

urn:lsid:zoobank.org:act:2DA7CCAE-4DD2-4CF0-A722-88CA003C691A

http://species-id.net/wiki/Chlaenius_(Lithochlaenius)_chuanqianensis

Figs 16–17 52 66 80 94–95 117–118 146, 150 158 

#### Types.

 Holotype: male (IZCAS), "China, Guizhou, Xishui County, Dabaitang, 600m”/ "IOZ & Guizhou Univ. Joint Expedition, 2000.9.24, Liang H.B” / ""Holotype, *Chlaenius (Lithochlaenius) chuanqianensis* Liu & Liang sp. n."" [red label]. Paratypes: Total 14 specimens. 1 female (IZCAS), "China, Guizhou, Xishui County, Dabaitang, 550m”/ "IOZ & Guizhou Univ Joint Expedition, 2000.9.27, Liang H. B.”; 1 female (HBUM), "China, Guizhou, Xishui County, Dabaitang”/ "2000. IX. 25–29, collector Ren G. D.”; 1 male (IZCAS), "China, Guizhou, Xishui County, Chengzhai, Hongqi village, 28.419033, 106.273766”/ "2009.10.8, Liu Y. & Shi H. L.”; 1 female (IZCAS), "Sichuan, Ya-an”; / ""1990.VII.3, Xie Weiping collector”; 1 male (IZCAS), "Sichuan, Xinjin” / ""1981.VI.13, Liu Hongjiang collector”; 1 female (IZCAS), "Sichuan, Anxian”; 1 male (IZCAS), "Sichuan, Shehong” / ""1980.7.7”; 3 males and 3 females (IZCAS), "Sichuan, Yajiang, Hekou Town, Shanbeihou, Yalongjiang, N30.00020, E101.01526” / "2009.5.28 N, Liang Hongbin coll.”; 1 female (IZCAS), "Sichuan, Yajiang, Hekou Town, Shanbeihou, Yalongjiang, E30.00020, N101.01526”/ "2009.5.27, Day, Liang Hongbin coll.”. Each paratype with an additional yellow label: ""Paratype, *Chlaenius (Lithochlaenius) chuanqianensis* Liu & Liang sp. n."".

#### Diagnosis.

Antennomere 1 coniform (Fig. 52); color of antennomere 1 yellow or brown; intervals 1–5 glabrous medially, with one row of pubescence laterally (Fig. 80); aedeagus stout, apical lamella round (Fig. 117), thin and straight in lateral view (Fig. 118).

#### Description.

Total length = 16.0–17.0 mm (mean = 16.8), width = 5.60–6.13 mm (mean = 5.86); HW = 2.60–2.95 mm (mean = 2.82), EYL = 0.95–1.10 mm (mean = 1.03), ratio Ant3/Ant1 = 1.56–1.72 (mean = 1.64), PL/PW = 0.76–0.84 (mean = 0.81), EL/EW = 1.68–1.91 (mean = 1.79), EL/ PL = 1.37–1.49 (mean = 1.42).

Head and pronotum black with green or coppery metallic luster; elytra black; ventral surface black; mandibles and trochanters dark brown; antennomere 1, femora, tibiae yellow to brown; antennomeres 2–11, palpi and tarsi dark brown to nearly black.

Head with vertex smooth medially, coarsely punctate behind eyes; labrum slightly emarginate at apex; antennomere 1 coniform (Fig. 52). Pronotum with disk smooth, very sparsely punctate along midline in a few specimens; lateral furrow sparsely pubescent and coarsely punctate; medial longitudinal furrow deep, impunctate; basal foveae narrow, deep, sparsely pubescent and punctate. Elytral intervals convex, intervals 1–5 glabrous medially, with a row of setae laterally (Fig. 80), pubescent in apical one-fifth in a few specimens, intervals 6–9 pubescent throughout; striae deep, punctate; humeral angles obtuse (Fig. 66). Abdominal sterna IV–VI sparsely pubescent medially, densely pubescent laterally; sternum VII narrowly rounded apically in both sexes (Figs 94–95). Lamella of aedeagus round at apex (Fig. 117), thin and straight in lateral view (Fig. 118).

#### Etymology.

 The Latinized name *chuanqianensis* refers to type localities of this new species in "chuanqian” regions, of which "chuan” refers to Sichuan Province and "qian” refers to Guizhou Province).

#### Geographical distribution.

 Fig. 158. Known only from Guizhou and Sichuan Provinces, China.

#### Remarks.

 Mensural data cited in the description were obtained from the holotype and all paratypes.

Members of this species are most similar to those of *Chlaenius wrasei* in shape of antennomere 1 and elytral pubescence, but differ from the latter in color of antennomere 1 (paler than antennomere 3 in *Chlaenius chuanqianensis*, antennomeres 1 and 3 concolorous in *Chlaenius wrasei*) and orientation of the apical lamella of the aedeagus of males (straight in *Chlaenius chuanqianensis*, bent ventrally in *Chlaenius wrasei*).

They are also similar to *Chlaenius leishanensis* members in pubescence of elytral intervals, but different from the latter in having yellow tibiae (black or dark brown in *Chlaenius leishanensis*), and males have a thin aedeagal lamella of the aedeagus (lamella thick in *Chlaenius leishanensis* males).

### Chlaenius (Lithochlaenius) linwensini

Liu & Liang sp. n.

urn:lsid:zoobank.org:act:EE7D65D7-2C84-497B-AA28-F62D7DACD5E7

http://species-id.net/wiki/Chlaenius_(Lithochlaenius)_linwensini

Figs 18–19 53 67 81 96–97 119–120 158 

#### Types.

Holotype: male (IZCAS), "Fujian, Jianyang, Huangkeng, Guiling, 270–340m""/ ""1960.III.26, Zhang Yiran collector""/ ""Holotype, *Chlaenius (Lithochlaenius) linwensini* Liu & Liang sp. n.""[red label]. Paratypes: Total 6 specimens. 1 male (IZCAS), "Fujian: Jianyang, Huangkeng, Guiling, 270–340m""/ ""1960.III.26, Zhang Yiran collector""; 1 female (IZCAS), "Fujian, Chong"an, Xingcun Sangang, 740m, light trap""/ "1960.VII.15, Zhang Yiran collector""; 1 female (IZCAS), "Fujian: Chong-an, San-gang""/ "1979.VIII.5, Song Shimei collector""; 1 male (SIECAS), "Fujian, Chong"an, Xingcun""/ "1960.VI.27, Jin Lin collector""; 1 male (SIECAS), "Fujian: Chong"an, Xingcun""; 1 male (IZCAS), "Fujian: Dehua, Chenguan, 510–550m""/ ""1960.VI.2, collector Ma Chenling"". Each paratype with an additional yellow label: "Paratype, *Chlaenius (Lithochlaenius) linwensini* Liu & Liang sp. n."".

#### Diagnosis.

Antennomere 1 coniform (Fig. 53); all antennomeres concolorous; basal two-thirds of intervals 1–5 glabrous medially, with irregular setae laterally (Fig. 81); lamella of aedeagus subtruncate at apex (Fig. 119), thickened and bent ventrally (Fig. 120).

#### Description.

Total length = 14.50–16.50 mm (mean = 15.71), width = 5.33–5.87 mm (mean = 5.60); HW = 2.40–2.85 mm (mean = 2.71), EYL = 1.00–1.15 mm (mean = 1.05), ratio Ant3/Ant1= 1.37–1.61 (mean = 1.50), PL/PW = 0.82–0.90 (mean = 0.86), EL/EW = 1.67–1.81 (mean = 1.76), EL/ PL = 1.27–1.41 (mean = 1.35).

Head and pronotum black, with green or coppery metallic luster; elytra black; ventral surface black; antennae, palpi, femora, and tibiae brown; mandibles, trochanters, and tarsi dark brown.

Head with vertex smooth medially, coarsely punctate and sparsely pubescent behind eye; labrum slightly emarginate at apex; antennomere 1 strongly coniform (Fig. 53). Pronotum with disk smooth medially, very sparsely punctate and pubescent along the middle line; lateral furrow very sparsely punctate; basal foveae small, deep, coarsely punctate and sparsely pubescent. Elytral intervals convex, basal two-thirds of intervals 1–5 glabrous medially, with irregularly arranged setae laterally (Fig. 81), apical one-third of intervals 1–5 sparsely pubescent medially, intervals 6–9 densely pubescent; striae deep, punctate; humeral angle obtuse (Fig. 67). Abdominal sterna IV–VI sparsely pubescent medially, densely pubescent laterally; sternum VII broadly rounded apically in male, narrowly truncate apically in female (Figs 96–97). Lamella of aedeagus truncate at apex (Fig. 119), thick and bent ventrally (Fig. 120).

#### Etymology.

 The Latinized name*linwensini* refers to Mr. Lin Wensin, an excellent insect collector who died during a recent collecting trip to Hainan, China.

#### Geographical distribution.

Fig. 158. Known only from Fujian Province, China.

#### Remarks.

 Mensural data cited in the description were obtained from the holotype and all paratypes.

Male members of this species are similar to those of *Chlaenius formosensis* in the shape of the lamella of aedeagus, but males and females differ from those of the latter in having antennomere 1 coniform (cylindrical in *Chlaenius formosensis*), elytral intervals 6–7 pubescent throughout (glabrous medially in *Chlaenius formosensis*), and males have the aedeagus convex ventrally in the middle portion (straight in *Chlaenius formosensis* males).

### Chlaenius (Lithochlaenius) propeagilis

Liu & Kavanaugh sp. n.

urn:lsid:zoobank.org:act:0DB721CA-0192-49D6-BD14-A9320D1D574C

http://species-id.net/wiki/Chlaenius_(Lithochlaenius)_propeagilis

Figs 14–15 20–21 54 68 82 98–99 121–122 148 158 

#### Types.

 Holotype: male (IZCAS),"Southwest Yunnan, Gongshan, Dulongjiang, 0.5km N of Dizhengdang, 28.08442, 98.32652”/ "1880 m, 2004.10.29, David Kavanaugh Coll.”/ "Holotype *Chlaenius (Lithochlaenius) propeagilis* Liu & Kavanaugh sp. n.” [red label]. Paratypes: Total 452 specimens (IZCAS, CASC): 1 male, "China, Yunnan Provin. Gongshan County, Cikai town, along street, N27°44'43", E98°39'53"”/ "1500 m, 2006.5.5, Liang H.B, Ba Weidong”; 2 males, "China, Yunnan, Gongshan County, Cikai Township, Nu Jiang at Dashaba, N27.73845, E098.67092”/ "1430 m, 8–9 October 2002, Stop #DHK 2002–40, D.H. Kavanaugh, P.E. Marek & H.B. Liang collectors”; 17 males and 11 females, "China, Yunnan Provin. Gongshan County, Cikai town, Pulahe, N27°46'08", E98°39'12"”/ "1510 m, 2002.9.21–24, Liang Hongbin, Ba Weidong”; 1 male and 1 female, "China, Yunnan, Gongshan, Cikai Township, 3.3 airkm NW of Gongshan above hydropower diversion dam, 1530 m”/ "N27.77175, E098.64924, 24 September 2002, Stop # DHK 2002–028, D.H. Kavanaugh collector”; 2 males, "China, Yunnan Prov. Gongshan, Cikai Town, Pulahe joint of Nujiang, 27.74843N, 98.66498E”/ "1530m, 2004.10.23, D. Kavanaugh, Dong D.Z.”; 6 males and 6 females, "China, Yunnan Provin. Gongshan County, Cilou (Power Station), N27°46'14", E98°39'16"”/ "1510m, 2002.5.6, Liang H.B, Ba Weidong, Yang Guodong, Li X.Q”; 5 males and 3 females, "China, Yunnan Provin. Gongshan County, Cikai town, Gazu Station, N27°44'35", E98°36'17"”/ "1600–1750 m, 2002.5.5, Liang H.B, Ba Weidong”; 2 males, "China, Yunnan Provin. Gongshan County, Cikai town to Qiqi Station, N27.43086, E98.34150”/ "1700–2000m, 2002.4.29, Liang Hongbin, Ba W.D.”; 7 males and 26 females, "China, Yunnan, Gongshan, Cikai Township, 3.0 airkm N of Gongshan on Pula He at hydropower diversion dam, 1500 m”/ "N27.77055°, E098.65446°, 24 September 2002, Stop # DHK 2002–027, D.H. Kavanaugh, P.E. Marek & D.Z. Dong collectors”; 3 males and 4 females (CASC), "CHINA, Yunnan Province, Gaoligong Shan, Nujiang Prefecture, Gongshan County, Qiqi He just above hydroelectric plant, 1500m”/ "N27.75748°, E98.66073°, 22 July 2000, Stop #00–269, D.H. Kavanaugh, Liang H.-B., & Dong D.-Z. collectors”; 2 males and 4 females, "China, Yunnan, Gongshan, Bingzhongluo Township, 34km N of Gongshan at junction of Shuangla He and Nu Jiang, 1550m”/ "E27.96918°, E098.66198°, 25 September 2002, stop # DHK 2002–039, D.H. Kavanaugh, P.E. Marek & D.Z. Dong collectors”; 4 males and 6 females, "China, Yunnan Prov. Gongshan, Bingzhongluo, Shuangla He, beach, 27.96817N, 98.66187E”/ "1520 m, 2004.10.22, D. Kavanaugh collector”; 12 males and 10 females, "China, Yunnan, Gongshan, Bingzhongluo, Shuanglahe, riverside, N27°58'59", E98°39'15"”/ "1588 m, 2002.9.22–26, Liang H.B. Li Xiangqian”; 2 males and 5 females, "China, Yunnan, Gongshan, Bingzhongluo, Shuanglahe, riverside, N27°58'59", E98°39'15"”/ "1588 m, 2002.7.20, Ba Weidong”; 5 males and 2 female, "China, Yunnan Prov. Gongshan, Dulongjiang, 0.5 km N of Dizhengdang, 28.08442N, 98.32652E”/ "1880 m, 2004.10.29; D. Kavanaugh, Dong D.Z.”; 7 males and 4 females, "China, Yunnan Provin. Gongshan County, Dulongjiang, Kongdang, headlamp, 27.87764N, 98.33618E”/ "1510m, 2006.8.27, David Kavanaugh”; 12 males and 17 females, "China, Yunnan Provin. Gongshan, Dulongjiang, Xianjiudang village, 27.94092N, 98.33340E”/ "1580m, 2004.11.4, D. Kavanaugh, Dong D.Z.”;1 male, "China, Yunnan, Fugong County, Shangpa Town, west bank of Nu Jiang, 1185 m, N26.90668, E098.86339”/ "13 October 2002, Stop #DHK 2002–047, D.H. Kavanaugh, P.E. Marek & H.B. Liang, D.Z. Dong collectors”; 5 males and 3 females, "China, Yunnan Prov. Fugong, Shangpa Town, Nujiang, River, 26.90650N, 98.86397E”/ "1175 m, 2004.4.20, Liang H.B. coll.”; 9 males and 4 females, "China, Yunnan Prov. Fugong, Shangpa Town, Beach of Nujiang, 26.90650N, 98.86397E”/ 1175 m, 2005.8.20, Liang H.B. Zhang J.F. Dong D.Z.”; 7 males and 5 females, "China, Yunnan Prov. Fugong, Shangpa Town, Mugujia, riverside, 26.86203N, 98.87142E”/ 1177 m, 2005.8.22, Dong Dazhi collector”; 1 male and 2 females, "China, Yunnan Prov. Fugong, Shangpa, Mugujia, round waterfall, 26.86203N, 98.87142E”/ 1177 m, 2005.8.22, Liang H.B. Zhang J.F.”; 1 female, "China, Yunnan, Fugong, Lumadeng Township, 2km airkm S of Aludi on Nu Jiang, 1245 m, N27.09037, E098.87359”/ "20 September 2002, Stop # DHK 2002–022, D.H. Kavanaugh & H.B. Liang collectors”; 1 female, "China, Yunnan Prov., Fugong, Pihe at junction of Nujiang River, 26.53177N, 98.89753E”/ "1060 m, 2004.4.20, D. Kavanaugh, C. Griswold”; 2 males and 6 females, "China, Yunnan, Lushui, Liuku, west bank of Nu Jiang, 960 m, N25.854, E098.852/ "15 October 2002, stop # DHK 2002–050, D.Z. Dong collector”; 1 male and 2 females, "China, Yunnan Provin. Lushui, Beach of Nujiang River, under stone, N25°51'20", E98°50'58"”/ 800 m, 2002.9.19, Liang Hongbin”; 2 males and 2 females (CASC), "CHINA, Yunnan Province, Gaoligongshan Mountains, Nujiang Prefecture, Lushui County, Salween River, 17 km N of Liuku, 970m”/ "25°58.7'N, 98°50.4'E, 21 October 1998, Stop #98–120, D.H. Kavanaugh & C.-L. Long collectors”; 2 females (CASC), "CHINA, Yunnan Province, Nujiang Prefecture, Lushui County, Liuku Township, Liuku, 800m”/ "25.86010°N, 98.85155°E, 25–26 June 2000, Stop #00–7, D.H. Kavanaugh & Liang H.-B. collectors”; 4 males and 5 females (CASC), "CHINA, Yunnan Province, Nujiang Prefecture, Lushui County, San jiang Township, Nu Jiang, 790m”/ "25.72964°N, 98.87180°E, 26 June 2000, Stop #00–9, D.H. Kavanaugh & Liang H.-B. collectors”; 7 male and 7 females, "China, Yunnan Prov. Baoshan, Longyang, Bawan, Dongfengqiao, 24.98742N, 98.87047E”/ "670 m, 2005.6.1, D. Kavanaugh, Dong D.Z.”; 15 male and 16 females, "China, Yunnan Prov. Baoshan, Longyang, Bawan, Dongfengqiao, 24.98535N, 98.87382E”/ "670 m, 2005.5.29–6.1, Liang H.B. Dong D.Z.”; 1 male, "China, Yunnan Prov. Tengchong, Mangbang, Longwenqiao, 25.02329N, 98.67710E”/ "1290 m, 2006.6.5, Liang H.B. Hu P.”; 6 males and 7 females, "China, Yunnan Prov. Tengchong, Mangbang, Longwenqiao, beach, 25.02396N, 98.67675E”/ "1285 m, 2006.6.5, David Kavanaugh”; 3 males and 1 female, "China, Yunnan Province, Tengchong, Shangying, N25°02'29.7", E98°40'22.9"” / "1335 m, 2003.10.19, Liang H.B, Shi X.C.”; 2 males and 8 females, "China, Yunnan Prov. Tengchong Co., Wuhe Town, Longjiangqiao, 24.89499N, 98.67510E”/ "1205 m, 2005.V.24, Kavanaugh D. Dong D.Z.”; 1 female, "China, Yunnan Province, Tengchong, Wuhe Township, Longjiang Bridge on Longchuanjiang”/ "N24.89889, E098.67667, 1215 m, 28 October 2003, under rocks, Dong D.Z. collector”; 17 males and 9 females, "China, Yunnan Prov. Tengchong, Wuhe, Longjiangqiao, beach, 24.89176N, 98.67551E”/ "1230 m, 2006.6.3, Kavanaugh D. Brett R.”; 4 males and 4 females, "China, Yunnan Prov. Tengchong Co., Wuhe Town, Longjiangqiao, 24.89284N, 98.67439E”/ "1210 m, 2005.V.24, Liang H.B. Yang J.L”; 1 male, "China, Yunnan Prov. Tengchong, Wuhe, Longjiangqiao, beach, 24.89293N, 98.67489E”/ "1220 m, 2006.6.3, Liang H.B. Hu P.”; 3 males, "China, Yunnan Province, Tengchong Co. Qushi Township, Shuang He Cun, N25.32555, E098.60861”/ "1464 m, 21 October 2003, under rocks, Dong D.Z. collector”; 2 males, "China, Yunnan Prov. Tengchong, Qushi, Xiangyangqiao, beach, 25.21221N, 98.57836E”/ "1500 m, 2006.V.24, David Kavanaugh”; 2 females, "China, Yunnan Prov. Tengchong, Qushi, Xiangyangqiao, beach, 25.23939N, 98.62723E”/ "1440 m, 2006.V.24, David Kavanaugh”; 11 males and 14 females (CASC), "CHINA, Yunnan Province, Gaoligongshan Mountains, Baoshan Prefecture, Tengchong County, Longchuan River at Longkou village”/ ”25°16.9'N, 98°35.5'E, 1500 m, 25 October 1998, Stop #98–126, D.H. Kavanaugh, C.E. Griswold, & C.-L. Long collectors”; 6 males and 1 female, "China, Yunnan Prov. Tengchong, Qushi, Longkou, beach, 25.28175N, 98.59246E”/ "1500 m, 2006.6.6, David Kavanaugh”; 5 males and 1 female (CASC), "CHINA, Yunnan Province, Gaoligongshan Mountains, Baoshan Prefecture, Tengchong County, Longchuan River at Longkou village”/ "25°16.9'N, 98°35.5'E, 1500 m, 2 November 1998, Stop #98–128, D.H. Kavanaugh, C.E. Griswold, C.-L. Long, R. Li, & H.-X. He collectors”; 3 males and 5 females, "China, Yunnan Prov. Tengchong, Qushi, Qinqiao, 25.27236N, 98.60093E”/ "1460 m, 2006.6.2–6, David Kavanaugh, Brett R.”; 4 males and 4 females, "China, Yunnan Province, Tengchong, Zhoujiapo Village, N25.33222, E098.67611”/ "1740 m, 24 October 2003, under rocks Dong D.Z. collector”; 3 males, "China, Yunnan Prov. Tengchong, Jietou, Yonganqiao, beach, 25.32502N, 98.70459E”/ "1470 m, 2006.V.24, Liang H.B.”; 2 males and 1 female, "China, Yunnan Prov. Tengchong, Jietou, Yonganqiao, beach, 25.32504N, 98.60959E”/ "1470 m, 2006.5.24, Kavanaugh D. Brett R.”; 1 male, "China, Yunnan Prov. Tengchong, Hehua, Dengma, on beach, 24.92346N, 98.38612E”/ "1105 m, 2006.6.2, Kavanaugh D. Brett R.”; 3 males and 1 female, "China, Yunnan Province, Jingping Co., Mengla Town, Mengla-daqiao, River side 22˚39'45.7",103˚04'44.7"”/ "312m, 2003.12.15, day, Liang H B, Boris Kataev Colls.”; 12 males and 10 females (IZCAS), "Yunnan, Jingdong, 1100m”/ "1982.IV.29–V.2, Yu P.Y.& Liao S.B.”; 1 female, "Yunnan, Honghe Prefecture, Lvchun, Huanglianshan, 2009.V. 16, Bai X. X. coll.” 2 males and 1 female, "Yunnan, Menglun, Xishuanbanna Botanical Garden”, "2005.5.22, light trap, Zheng Guo leg.”; 3 males and 5 females, "China, Sichuan, Yajiang, Hekou Town, Shabeihou, Yalongjiang, N30.00020, E101.01526”/ "2583m, 2009.5.27–28, Liang H.B.”; 1 female (IZCAS), "China, Tibet, Bomi Yi"ong, Tangmai bridge, Beach of Yi"ong Zangbo, 30.09633N, 95.06577E”/ "2035m, 2006.8.30 N, Liang H.B., Song Z.S.”. Each paratype with an additional yellow label: "Paratype *Chlaenius (Lithochlaenius) propeagilis* Liu & Kavanaugh sp. n.”

#### Diagnosis.

Antennomere 1 elongate ovoid (Fig. 54); color of antennomeres 1–3 lighter than antennomeres 4–11; intervals 1–5 almost glabrous medially, with one or two rows of pubescence at each lateral side, more evenly pubescent near apex (Fig. 82); apical lamella of aedeagus moderately triangular (Fig. 121), thick and reflexed in lateral view (Fig. 122).

#### Description.

Total length = 15.50–17.00 mm (mean = 15.71), width = 5.60–6.13 mm (mean = 5.81); HW = 2.75–2.95 mm (mean = 2.82), EYL = 1.00–1.15 mm (mean = 1.09), ratio Ant3/Ant1 = 1.73–1.93 (mean = 1.81), PL/PW = 0.78–0.85 (mean = 0.83), EL/EW = 1.62–1.79 (mean = 1.73), EL/ PL = 1.29–1.45 (mean = 1.36).

Head and pronotum black with green or coppery metallic luster; elytra black; ventral surface black, with some slight metallic reflection; coxae almost black; mandibles dark brown; antennomeres 4–11, palpomere, trochanters, tibiae at both ends, and tarsomeres reddish or brown; antennomeres 1–3, femora and middle portions of tibiae yellow or yellowish-brown.

Head rugose near eyes and occiput, vertex sparsely rugose; eyes prominent; antennomere 1 elongate–ovoid (Fig. 54); labrum concave at apex, with distinct microsculpture; mentum tooth emarginate apically.

Pronotum cordate, moderately convex; lateral margins bordered; disk almost smooth, sparsely rugose and setose near base and lateral margins; basal foveae moderately deep.

Elytra widest at mid-length; humeral angle obtuse (Fig. 68); striae deep, punctate; intervals moderately convex, intervals 1–5 almost glabrous medially, with a row of pubescence laterally, intervals more evenly pubescent near apex (Fig. 82); intervals 6–9 pubescent throughout and with intervals 7–9 more densely pubescent than 6.

Abdominal sterna bordered, densely pubescent laterally; sterna IV–VI sparsely pubescent medially; sternum VII rugose–pubescent, with apex narrowly rounded or subobtuse in males (Fig. 98), more broadly rounded in female (Fig. 99).

Apical lamella of aedeagus moderately triangular (Fig. 121), thick and bent ventrally (Fig. 122). Gonostylus (Figs 14–15).

#### Etymology.

 The Latinized name*propeagilis* refers to the similarity of members of this species to those of *Chlaenius agilis*.

#### Geographical distribution.

Fig. 158. Known only from southeastern Tibet and Yunnan Province, China.

#### Remarks.

 Mensural data cited in the description were based on measurements obtained from 5 males and 5 females selected for maximum variation.

Specimens of *Chlaenius (Lithochlaenius)* collected from western Yunnan were initially determined as *Chlaenius agilis* Chaudoir. However, after comparison of the male genitalia with those of type specimens of *Chlaenius agilis*, we are convinced that they represent a distinct new species. Males differ from those of *Chlaenius agilis* in having the median lobe of aedeagus gradually bent near the base in lateral view (Fig. 122), whereas *Chlaenius agilis* males have the median lobe abruptly bent with a depression near the base (Fig. 124).

This species is clearly very closely related to *Chlaenius agilis*. At present, the known ranges of these two species are broadly disjunct (Fig. 158). We know of no locality records for any *Chlaenius (Lithochlaenius)* species from the intervening area (i.e., between northcentral India and southeastern Tibet and western Yunnan Province. Whether this distributional gap represents a real disjunction or is only an artifact of inadequate collecting in the area to date can only be determined from additional sampling efforts in the region. It would be particularly informative to determine whether or not any populations representing the *propeagilis/agilis* lineage occur in the region and, if so, whether or not males display intermediate genitalic traits.

### Chlaenius (Lithochlaenius) agilis

Chaudoir

http://species-id.net/wiki/Chlaenius_(Lithochlaenius)_agilis

Figs 22–23 36 41 46–49 55, 62–64 69, 76–78 83, 90–92 100, 113–115 123–124 138–143 147 158

Chlaenius latro  LaFerté-Sénectère, 1851:250 (unavailable). Nomen nudum **stat. n.**
Chlaenius agilis  Chaudoir, 1856:246; 1876:193; Kryzhanovskij, 1976:12
Chlaenius anchomenoides  Bates, 1889:212; Kirschenhofer, 1997:116; Kirschenhofer, 2005:490. **syn. n.**
Chlaenius nuristanus  Jedlička, 1956:194; Kirschenhofer, 2005:490. **syn. n.**
Chlaenius nuristanus  a. *rubridipes* Jedlička, 1956:194 (not available, ICZN Articles 10A and 45.6.2).
Stenochlaenius anchomenoides  Bates: Mandl, 1972:104

#### Specimens examined.

 Total 15 specimens. **India:** type: male (MNHN), "*agilis* Chaud, Ind. orient bor. C. Boys.”/ "TYPE *agilis*”/ "Ex Musaeo Chaudoir”/ "agilis Chd”; Cotype: female (MNHN), "*agilis* Chaud, Ind. orient bor. C. Boys.”/ "TYPE agilis”/ "Ex Musaeo Chaudoir”/ "agilis Chd” ; Cotype: male (MNHN), "*agilis* Chaud, Ind. orient bor. C. Boys.”/ "*latro*”; 2 males and 3 females (CASC), "Bajaura, Kongra district (Indes Angl.)”/ "G. Babault, Juin 1914”/ "van Dyke collection”/ "*Chlaenius agilis*, Chaud., H.E. Andrewes det.”. 1 female (CASC), "W. Almora Divn, Kumaon U.P., Apr. 1917, HGC.”/ "Van Dyke Collection”. **Pakistan:** Cotype: 2 males (BMNH), "Goorais valley, 7000ft, V. 87”/ "H. E. Andrews Coll. B. M. 1945–97”/ "Ex coll. R. Oberthür”/ "*Chlaenius anchomenoides* Bates, cotype, H. E. Andrews det.”/ "Co-type”. **Afghanistan**: 1 female (MNHN), "J. Klapperich, Bashgultal, 1100m, Nuristan, 22.4.53, Afghanistan”/ "*Chlaenius nuristanus* sp. n, det. ING. JEDLICKA”/ "Type”;

Paratype: 2 males (MNHN), "J. Klapperich, Asmar, 900m, Kunartal, 3.4.53, O-Afghanistan”/ "*Chlaenius nuristanus* sp.n, det. ING. JEDLICKA”/ "PARATYPE”; Type: 1 male (MNHN), "J. Klapperich, Asmar, 900m, Kunartal, 3.4.53, O-Afghanistan”/ "*Chlaenius nuristanus* a. *rubridipes* n. det. ING. JEDLICKA”/ "TYPUS”; Cotype: 1 male (MNHN), "J. Klapperich, Asmar, 900m, Kunartal, 3.4.53, O-Afghanistan”/ "*Chlaenius nuristanus* a. *rubridipes* n. det. ING. JEDLICKA”/ "Cotype”.

#### Diagnosis.

Antennomere 1 elongate ovoid (Figs 55, 62–64); basal two-thirds of intervals 1–5 glabrous medially, with one row of pubescence laterally near striae (Figs 83, 90–92); aedeagus abruptly bent near base (Figs 124, 139, 141, 143).

#### Description.

Total length = 15.50–16.00 mm (mean = 15.50), width = 5.60–6.10 mm (mean = 5.73); HW = 2.75–2.95 mm (mean = 2.82), EYL = 1.00–1.15 mm (mean =1.00), ratio Ant3/Ant1 = 1.67–1.83 (mean = 1.71), PL/PW = 0.84–0.85 (mean = 0.85), EL/EW = 1.63–1.73 (mean = 1.71), EYL/ PL = 1.15–1.44 (mean = 1.34). Mensural data cited in the description were obtained from type and cotype specimens examined.

Head, pronotum and elytra black, with green or blue metallic luster; ventral surface black; mandibles and trochanters dark brown; palpomeres, femora, and tarsomeres brown to dark brown; antennae and tibiae yellow to brown (Figs 22–23, 36–41).

Head with vertex smooth or very sparsely punctate behind eyes; antennomere 1 elongate ovoid (Figs 55, 62–64); labrum slightly emarginate at apex. Pronotum with disk smooth; basal foveae deep, sparsely punctate and pubescent (Figs 22, 36, 38, 40). Elytra with intervals convex, basal two-thirds of intervals 1–5 glabrous medially, with one row of pubescence laterally near striae (Figs 83, 90–92), intervals 6–9 and apical one-third of intervals 1–5 densely pubescent; striae deep, punctate; humeral angle obtuse (Figs 69, 76–78). Abdominal sterna IV–VI sparsely pubescent medially; sternum VII rugose–pubescent, narrowly rounded apically in males (Figs 100, 113–114), broadly rounded apically in females (Fig. 115). Aedeagus abruptly bent near base, apical lamella round (Figs 123, 138, 140, 142), thin and slight bent ventrally (Figs 124, 139, 141, 143).

**Figures 46–51.**
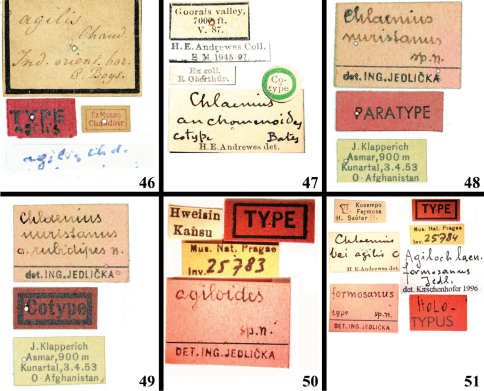
Labels of *Chlaenius (Lithochlaenius)* spp**46** holotype, *Chlaenius agilis* Chaudoir (see Figs 22–23)**47** Cotype, *Chlaenius anchomenoides* Bates (see Figs 40–41)**48** Paratype, *Chlaenius nuristanus* Jedlička (see Figs 42–43)**49** Cotype, *Chlaenius nuristanus* Jedlička (see Figs 44–45)**50** Holotype, *Chlaenius agiloides* Jedlička (see Fig. 48) **51** Holotype, *Chlaenius formosanus* Jedlička (see Fig 49).

**Figures 52–65.**
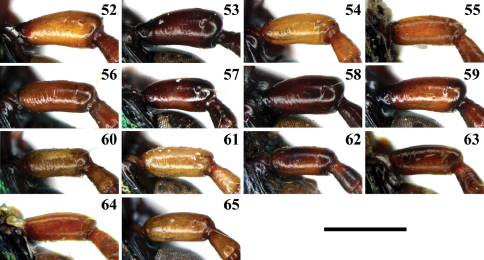
Antennomere 1 of *Chlaenius (Lithochlaenius)* species**52** *Chlaenius chuanqianensis* sp. n., holotype**53** *Chlaenius linwensini* sp. n., holotype**54** *Chlaenius propeagilis* sp. n., holotype**55** *Chlaenius agilis* Chaudoir, holotype**56** *Chlaenius formosensis* Lorenz, CCCC**57** *Chlaenius leishanensis* Kirschenhofer, IZCAS**58** *Chlaenius noguchii* Bates, IZCAS**59** *Chlaenius wrasei* Kirschenhofer, IZCAS**60** *Chlaenius agiloides* Jedlička, IZCAS**61** *Chlaenius rambouseki* Lutshnik from Ussuri region, IZCAS**62** *Chlaenius anchomenoides* Bates, paratype**63** *Chlaenius nuristanus* Jedlička, paratype**64** *Chlaenius nuristanus* a. *rubridipes* Jedlička, paratype**65** *Chlaenius rambouseki* Lutshnik from Taiwan,CCCC. Scale line = 1.0 mm.

**Figures 66–79.**
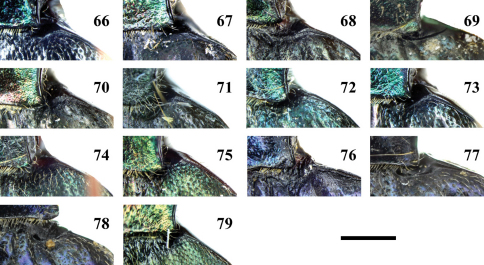
Humeral region of elytra of *Chlaenius (Lithochlaenius)* spp**66** *Chlaenius chuanqianensis* sp. n., holotype**67** *Chlaenius linwensini* sp. n., holotype**68** *Chlaenius propeagilis* sp. n., holotype**69** *Chlaenius agilis* Chaudoir, holotype**70** *Chlaenius formosensis* Lorenz, CCCC**71** *Chlaenius leishanensis* Kirschenhofer, IZCAS**72** *Chlaenius noguchii* Bates, IZCAS**73** *Chlaenius  wrasei* Kirschenhofer, IZCAS**74** *Chlaenius agiloides* Jedlička, IZCAS**75** *Chlaenius rambouseki* Lutshnik from Ussuri region, ZCAS**76** *Chlaenius anchomenoides* Bates, paratype**77** *Chlaenius nuristanus* Jedlička, paratype**78** *Chlaenius nuristanus* Jedlička, paratype**79** *Chlaenius rambouseki*Lutshnik from Taiwan, CCCC. Scale line = 1.0 mm.

**Figures 80–93.**
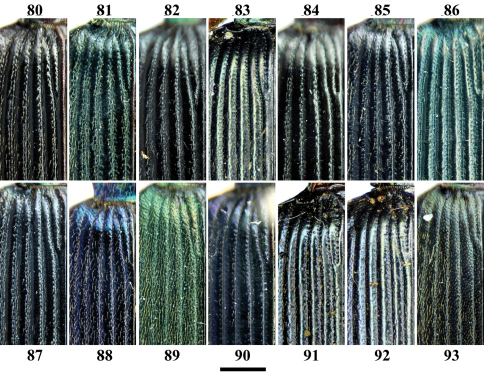
Elytral intervals of *Chlaenius (Lithochlaenius)* spp. **80** *Chlaenius chuanqianensis* sp. n., holotype**81** *Chlaenius linwensini* sp. n., holotype**82** *Chlaenius propeagilis* sp. n., holotype**83** *Chlaenius agilis* Chaudoir, holotype**84** *Chlaenius formosensis* Lorenz, CCCC**85** *Chlaenius leishanensis*, IZCAS**86** *Chlaenius noguchii* Bates, IZCAS**87** *Chlaenius wrasei* Kirschenhofer, IZCAS**88** *Chlaenius agiloides* Jedlička, IZCAS**89** *Chlaenius rambouseki* Lutshnik from Ussuri region, IZCAS**90** *Chlaenius anchomenoides* Bates, paratype**91** *Chlaenius nuristanus*Jedlička, paratype**92** *Chlaenius nuristanus* Jedlička, paratype**93** *Chlaenius rambouseki* Lutshnik from Taiwan*,*CCCC. Scale line = 1.0 mm.

**Figures 94–116.**
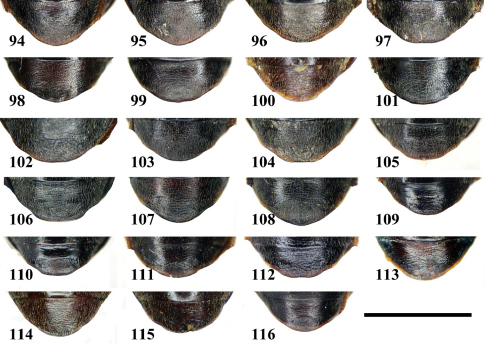
Abdominal sternum VII (ventral aspect) of *Chlaenius (Lithochlaenius)* species**94** *Chlaenius chuanqianensis* sp. n., male, holotype**95** *Chlaenius chuanqianensis* sp. n., female, paratype**96** *Chlaenius linwensini* sp. n., male, holotype**97** *Chlaenius linwensini* sp. n., female, paratype**98** *Chlaenius propeagilis* sp. n., male, holotype**99** *Chlaenius propeagilis* sp. n., female, paratype**100** *Chlaenius agilis* Chaudoir, male, holotype**101** *Chlaenius formosensis* Lorenz, male**102** *Chlaenius formosensis* Lorenz, female**103** *Chlaenius leishanensis* Kirschenhofer, male**104** *Chlaenius leishanensis* Kirschenhofer, female**105** *Chlaenius noguchii* Bates, male**106** *Chlaenius noguchii* Bates, female**107** *Chlaenius wrasei* Kirschenhofer, male**108** *Chlaenius wrasei* Kirschenhofer, female**109** *Chlaenius agiloides* Jedlička, male**110** *Chlaenius agiloides* Jedlička, female**111** *Chlaenius rambouseki* Lutshnik, from Ussuri region, male**112** *Chlaenius rambouseki* Lutshnik from Ussuri region, female**113** *Chlaenius anchomenoides* Bates, male, paratype**114** *Chlaenius nuristanus* Jedlička, male, paratype**115** *Chlaenius nuristanus* Jedlička, female, paratype**116** *Chlaenius formosanus* Jedlička from Taiwan, male. Scale line = 1.0 mm.

**Figures 117–130.**
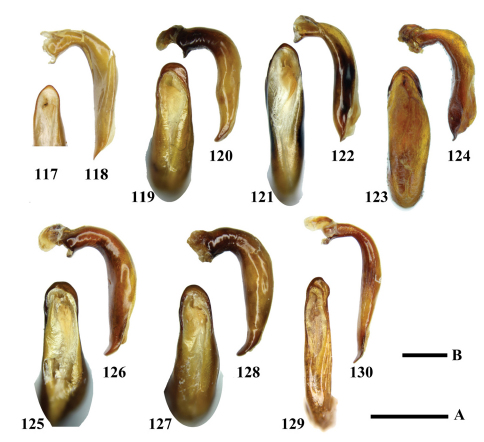
Aedeagi of *Chlaenius (Lithochlaenius)* species**117** *Chlaenius chuanqianensis* sp. n., holotype (dorsal view)**118** *Chlaenius chuanqianensis* sp. n., holotype (left lateral view)**119** *Chlaenius linwensini* sp. n., holotype (dorsal view)**120** *Chlaenius linwensini* sp. n., holotype (left lateral view)**121** *Chlaenius propeagilis* sp. n., holotype (dorsal view)**122** *Chlaenius propeagilis* sp. n., holotype (left lateral view)**123** *Chlaenius agilis* Chaudoir, holotype (dorsal view)**124** *Chlaenius agilis* Chaudoir, holotype (left lateral view)**125** *Chlaenius formosensis* Lorenz, holotype (dorsal view)**126** *Chlaenius formosensis* Lorenz, holotype (left lateral view)**127** *Chlaenius leishanensis* Kirschenhofer (dorsal view)**128** *Chlaenius leishanensis* Kirschenhofer (left lateral view)**129** *Chlaenius noguchii*Bates (dorsal view)**130** *Chlaenius noguchii*Bates (left lateral view). Scale lines: A = 1.0 mm (Figs 117, 119, 121, 123, 125, 127, 129); B = 0.5 mm (Figs 118, 120, 122, 124, 126, 128, 130).

**Figures 131–145.**
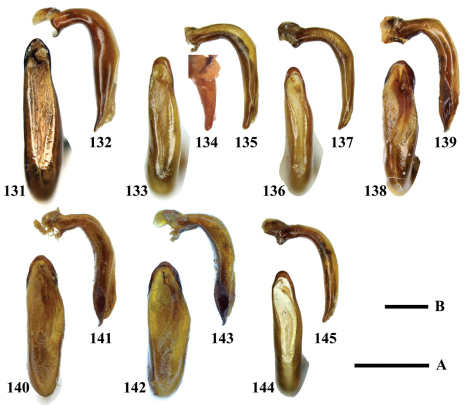
Aedeagi of *Chlaenius (Lithochlaenius)* species **131** *Chlaenius wrasei* Kirschenhofer (dorsal view) **132** *Chlaenius wrasei* Kirschenhofer (left lateral view)**133** *Chlaenius agiloides* Jedlička (dorsal view) **134** *Chlaenius agiloides* Jedlička, holotype (left lateral view) **135** *Chlaenius agiloides* Jedlička (left lateral view) **136** *Chlaenius rambouseki* Lutshnik from Ussuri region (dorsal view)**137** *Chlaenius rambouseki* Lutshnik from Ussuri region (left lateral view) **138** *Chlaenius anchomenoides* Bates, paratype (dorsal view)** 139** *Chlaenius anchomenoides* Bates, paratype (left lateral view)**140** *Chlaenius nuristanus* Jedlička, paratype (dorsal view, black leg) **141** *Chlaenius nuristanus* Jedlička, paratype (left lateral view)** 142** *Chlaenius nuristanus* Jedlička, paratype (dorsal view, yellow leg)**143** *Chlaenius nuristanus* Jedlička, paratype (left lateral view)**144** *Chlaenius rambouseki*Lutshnik from Taiwan (dorsal view)**145** *Chlaenius rambouseki*Lutshnik from Taiwan (left lateral view). Scale lines: A = 1.0 mm (Figs 131, 133, 136, 138, 140, 142, 144); B = 0.5 mm (Figs 132, 134, 135, 137, 139, 141, 143, 145).

#### Color variation.

 Color of antennae, palpomeres, femora, and tibiae varied from medium brown to dark brown or even black among different individuals (Figs 22–23, 36–41).

#### Geographical distribution.

Fig. 158. Known only from eastern Afghanistan, Pakistan, and northern India.

#### Remarks.

 Mensural data cited in the description were obtained from type and cotype specimens examined. The name *Chlaenius agilis* Chaudoir was once treated as a junior synonym of *Chlaenius latro* LaFerté–Sénectère (Csiki 1931, Kirschenhofer 1997). However, in his work, LaFerté–Sénectère (1851) did not provide a specific description of *Chlaenius latro*. This means that *Chlaenius latro* LaFerté–Sénectère is a *nomen nudum*, and therefore, unavailable.

Kirschenhofer (2005) considered *Chlaenius nuristanus*Jedlička to be a junior synonym of *Chlaenius anchomenoides* Bates. We have examined the types and/or cotypes of *Chlaenius agilis* Chaudoir, *Chlaenius anchomenoide*s, *Chlaenius nuristanus* and its aberration *Chlaenius nuristanus*a.*rubridipes*, and found no significant differences between them, except for variation in the color of antennae and legs. The aedeagi are abruptly bent near the base in all dissected males (Figs 124, 139, 141, 143).

Mandl (1972: 104) assigned *Chlaenius anchomenoides* to genus *Stenochlaenius*. Based on the pubescent elytral intervals and incomplete elytral basal margin in members of *Chlaenius anchomenoides*, we do not agree with this assignment.

### Chlaenius (Lithochlaenius) formosensis

Lorenz

http://species-id.net/wiki/Chlaenius_(Lithochlaenius)_formosensis

Figs 24–25 56 70 84 101–102 125–126 158

Chlaenius noguchii formosanus  Habu, 1965: 86 (*nec* Jedlička, 1935:5)
Chlaenius formosanus  Morita, 1993:161
Chlaenius formosensis  Lorenz, 1998:339 (replacement name); Kirschenhofer, 2005:491

#### Specimens examined.

 Total 24 specimens: **China:** 8 males and 12 females (CCCC), "Taiwan Prov., Hsinchu, Wufeng, 1996.08.03, Chen C.C. coll.”; 3 males and 1 female (CCCC), "Taiwan Prov., Hsinchu, Wufeng, 1998.4.11, Chen C.C. coll.

#### Diagnosis.

Antennomere 1 cylindrical (Fig. 56); antennomeres 1–3 brown; intervals 1–7 convex, glabrous medially, with one row of pubescence laterally (Fig. 84); apex of sternum VII rounded (Figs 101–102); lamella of aedeagus slightly truncate at apex (Fig. 125), thick, bent ventrally (Fig. 126).

#### Description.

Total length = 16.0–17.0 mm (mean = 16.45), width = 5.87–6.40 mm (mean = 5.99); HW = 2.90–3.00 mm (mean = 2.96), EYL = 1.10–1.15 mm (mean = 1.12), ratio Ant3/Ant1 = 1.71–1.88 (mean = 1.76), PL/PW = 0.81–0.88 (mean = 0.85), EL/EW = 1.68–1.86 (mean = 1.76), EL/ PL = 1.32–1.48 (mean = 1.39).

Head and pronotum black, with green or coppery metallic luster; elytra black; ventral surface black; mandibles, trochanters, and tarsomeres dark brown; antennae, palpomeres, femora, and tibiae yellow to brown.

Head with vertex nearly glabrous, very sparsely punctate and pubescent near eyes; Antennomere 1 cylindrical (Fig. 56); labrum slightly emarginate at apex. Pronotum with disk convex, nearly smooth, glabrous; basal foveae small, deep, finely punctate, sparsely pubescent. Elytra with intervals convex, intervals 1–7 glabrous medially, with a row of pubescence laterally near striae (Figs 24, 84), intervals 8 and 9 with decumbent pubescence throughout; striae deep, punctate; humeral angle obtuse (Fig. 70). Abdominal sterna IV-VI nearly glabrous medially, densely pubescent laterally; sternum VII broadly rounded at apex in both sexes (Figs 101–102). Aedeagus with lamella subtruncate at apex (Fig. 125), thick and bent laterally (Fig. 126).

#### Geographical distribution.

Fig. 158. Known only from Taiwan.

#### Remarks.

 Mensural data cited in the description were based on measurements obtained from 5 males and 5 females selected for maximum variation.

This species was first described as a subspecies of *Chlaenius noguchii*. Later, Morita (1993) upgraded it to a distinct species. However, the name *Chlaenius formosanus* was preoccupied by another *Chlaenius* species of Jedlička, and therefore Lorenz (1998) renamed it *Chlaenius formosensis*.

Kirschenhofer (2005: 491) considered *Chlaenius formosensis* Lorenz to be a junior synonym of *Chlaenius formosanus* Jedlička (= *Chlaenius rambouseki* Lutshnik, see below) with no comparison. Based on Habu"s original description (type unavailable according to Morita), named specimens of *Chlaenius formosensis* in Morita's collection (corresponding author and checked by HBL in 2009), and specimens in Chen Chanchin's collection, we treat *Chlaenius formosensis* as a distinct species. Its members differ from those of *Chlaenius rambouseki*in having the vertex of the head and pronotal disk glabrous (both sparsely punctate and pubescent in *Chlaenius rambouseki*), intervals 1–7 glabrous medially (wholly pubescent in *Chlaenius rambouseki*), and the male aedeagus stout (slender in *Chlaenius rambouseki*).

*Chlaenius formosensis* adults are similar to those of *Chlaenius noguchii* in elytra pubescence, but differ from the latter in having antennomere 1 cylindrical (coniform in *Chlaenius noguchii*), pronotal disk smooth (finely punctate in *Chlaenius noguchii*), and male aedeagus stout (slender in *Chlaenius noguchii*).

### Chlaenius (Lithochlaenius) leishanensis

Kirschenhofer

http://species-id.net/wiki/Chlaenius_(Lithochlaenius)_leishanensis

Figs 26–27 57 71 85 103–104 127–128 149 158

Chlaenius leishanensis  Kirschenhofer, 2005:490

#### Specimens examined.

 Total 14 specimens: **China:** 1 male and 1 female (IZCAS), "Guizhou Prov. Leigongshan, Fangxiang, 2005.6.2–3, 1000–1100m, Ge Deyan collector”; 1 female (IZCAS) "Guizhou Prov. Leigongshan, Fangxiang, 900m, 2005.5.31, Xu Fangling and Cao Lingzhen collectors”; 1 male (IZCAS), "Guizhou, Leigongshan, Xiaodanjiang, 920–970m, 2005.6.3, Yang Zaihua collector”; 1 female (HBUM), "Guizhou, Daozhen, Xiannvdong, 2004.8.24–26, Yang Xiujuan and Hua Huiran collector”; 1 female (HBUM), "Guangxi, Yuanbaoshan, Xiangfen, 2004.7.19, Yu Yang and Gao Chao collector”; 1 female (IZCAS), "Guangxi, Longsheng”/ "1980.VI.11 Song Shimei coll.”; 1 male and 2 females (IZCAS), "Guangxi, Yangshuo, 1980.10”; 1 male and 2 females (IZCAS), "China, Hubei Prov. Shennongjia, Honghua Riverside, light trap, 31°24'20”, 110°28'40””/ "835m, 2003.8.10, night, Liang Hongbin coll.”; 1 female (IZCAS), "Beibei” [Chongqing] / ""1940.VIII.10”.

#### Diagnosis.

Antennomere 1 coniform (Fig. 57); color of antennomere 1 lighter than other antennomeres; basal half of intervals 1–5 glabrous medially, with pubescence laterally (Figs 26, 85); lamella of aedeagus rounded at apex (Fig. 127), thickened and bent ventrally (Fig. 128).

#### Description.

Total length = 15.00–18.00 mm (mean = 16.50), width = 5.33–6.13 mm (mean = 5.78); HW = 2.55–3.00 mm (mean = 2.79), EYL = 0.95–1.10 mm (mean = 1.04), ratio Ant3/Ant1 = 1.53–1.73 (mean = 1.62), PL/PW= 0.80–0.89 (mean = 0.86), EL/EW = 1.64–1.89 (mean = 1.76), EL/ PL = 1.26–1.46 (mean = 1.35).

Head and pronotum black, with green or coppery metallic luster; elytra black; ventral surface black; antennomeres 2–11, palpomeres, trochanters, tibiae, and tarsomeres dark brown to black; antennae and femora yellow to light brown.

Head with vertex coarsely punctate and pubescent behind eyes; antennomere 1 coniform (Fig. 57); clypeus and labrum slightly emarginate at apex. Pronotum with disk smooth in most specimens, but sparsely punctate along middle line in a few specimens; basal foveae small, deep, pubescent, very sparsely punctate. Elytra with basal half of intervals 1–5 smooth and glabrous medially, pubescent laterally (Figs 26, 85), intervals 6–9 and apical portion of intervals 1–5 densely pubescent; striae deep, punctate; humeral angle rounded (Fig. 71). Abdominal sterna IV-VI sparsely pubescent medially, densely pubescent laterally, apex of sternum VII rounded in male (Fig. 103), nearly truncate in female (Fig. 104). Aedeagus stout, lamella rounded at apex (Fig. 127), thickened and bent laterally (Fig. 128).

#### Geographical distribution.

Fig. 158. Known from Guangxi, Guizhou, Chongqing and Hubei Provinces, China.

**Figures 146–151.**
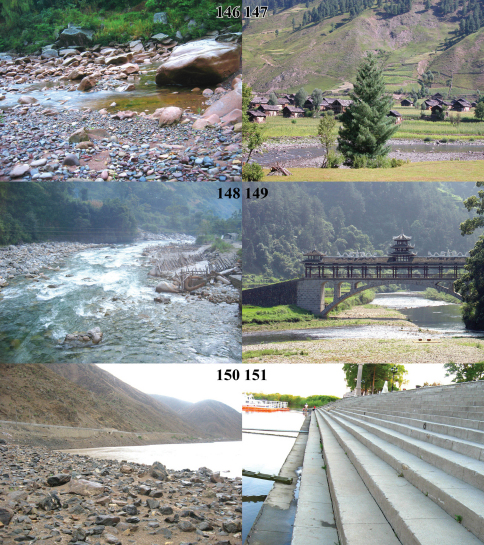
Photographs of habitats for *Chlaenius (Lithochlaenius)* spp. **146** *Chlaenius chuanqianensis* sp. n., Dabaitang, Xishui county, North Guizhou, China**147** *Chlaenius anchomenoides* Bates, Goorais valley, Pakistan (Provided by Dr. Muhammad Abbas in Pakistan Museum of Natural History)**148** *Chlaenius propeagilis* sp. n., Gaoligongshan, Yunnan, China**149** *Chlaenius leishanensis* Kirschenhofer,Xiaodanjiang, Leigongshan, Leishan county, Southeast Guizhou, China**150***Chlaenius chuanqianensis* sp. n. and *Chlaenius agiloides* Jedlička, Jinshajiang, Sichuan**151** *Chlaenius rambouseki* Lutshnik, Ussri river, Heilongjiang, China.

**Figures 152–157.**
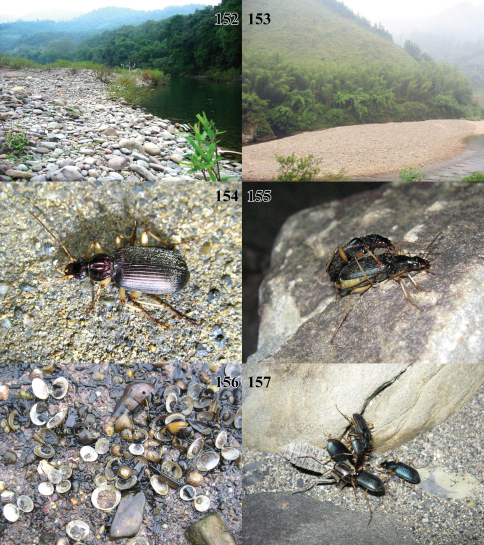
Photographs of habitats for *Chlaenius (Lithochlaenius)* spp. **152** *Chlaenius rambouseki* Lutshnik, Baisha county, Hainan Island, China**153** *Chlaenius rambouseki* Lutshnik, Yuanbaoshan, Guangxi, China**154** *Chlaenius rambouseki* Lutshnik adult walking on concrete wharf of Ussri river at night**155** *Chlaenius agiloides* Jedlička adults mating at night**156** Assorted molluscs, show one food of *Chlaenius rambouseki* Lutshnik from Hainan Island**157** Adults of *Chlaenius agiloides* Jedlička preying on dragonfly nymph at night.

#### Remarks.

 Mensural data cited in the description were obtained from all cited specimens.

Males of this species (Fig. 128) are similar to those of *Chlaenius formosensis*(Fig. 126) in form of the aedeagus in lateral view, but differ from the latter in having the lamellar apex rounded (Fig. 127; nearly truncate in *Chlaenius formosensis*, Fig. 125). Both males and females of *Chlaenius leishanensis* have elytral intervals 6–7 densely pubescent throughout, whereas *Chlaenius formosensis* adults have those intervals glabrous medially.

### Chlaenius (Lithochlaenius) noguchii

Bates

http://species-id.net/wiki/Chlaenius_(Lithochlaenius)_noguchii

Figs 28–29 58 72 86 105–106 129–130 158

Chlaenius noguchii  Bates, 1873:251; Chaudoir, 1876:192; Kryzhanovskij, 1976:12; Kirschenhofer, 1997:116; Kirschenhofer, 2005:491

#### Specimens examined.

 Total 47 specimens: **Japan:** 10 males and 11 females (IZCAS), "Shiga, Takashima, Biwa Lake, 2008.3.30, Liang H.B. coll.”; 1 male and 1 female (IZCAS), "Yadorigi-zawa, Tanzawa, Kanagawa”; 1 male (IZCAS), "Sado-shima Island”/ "1968.VIII.20”; 2 males and 2 females (CASC), "Harima Japan May 1916”; 1 female (CASC), "Hozukyo Kyoto 1951.9.21”; 4 males and 2 females (CASC); "Kobe V-1912”/ "L. Gressitt Collector”; 1 female (CASC), "Kyoto, Yase, Oct. 21, 1951, Col. T. Horio”; 2 females (CASC), "Mimasaka Japan VII. J. E. Lewis”; 2 males and 2 females (CASC), "Mimasaka Japan VII-1912”/ "Coll. by J. C. Thompson”; 1 male and 1 female (CASC), " Nikko Japan VII.30.23”/ "ECVan Dyke collector”; 2 females (CASC), "Nikko Hondo Japan VII-1912”/ "Coll. by J. C. Thompson”; 1 female (CAS), Tokyo, Japan VI-6-31.”/ "L. Gressitt Collector”.

#### Diagnosis.

Antennomere 1 coniform (Fig. 58); intervals 1–7 strongly convex, carinate, glabrous medially (Fig. 86).

#### Description.

Total length = 14.50–16.00 mm (mean = 15.33), width = 5.07–5.60 mm (mean = 5.33); HW = 2.50–2.90 mm (mean = 2.67), EYL = 1.00–1.10 mm (mean = 1.02), ratio Ant3/Ant1 = 1.56–1.88 (mean = 1.72), PL/PW = 0.83–0.90 (mean = 0.86), EL/EW = 1.75–1.91 (mean = 1.80), EL/ PL = 1.29–1.40 (mean = 1.35).

Head and pronotum black with strong green or bluish green metallic luster; elytra black with very weak blue luster; ventral surface black; legs, antennae, mandibles and palpomere dark brown.

Head with vertex coarsely punctate behind eyes; antennomere 1 coniform (Fig. 58); clypeus and labrum nearly truncate at apex; mentum tooth emarginate. Pronotum with disk finely punctate, nearly glabrous; basal foveae deep, finely pubescent and punctate (punctations much smaller than those on vertex). Elytra with intervals strongly convex, intervals 1–7 carinate, smooth and glabrous medially, with a row of punctation laterally (Fig. 86), intervals 8–9 flat, densely pubescent and punctate; striae deep, punctate; humeral angle obtuse (Fig. 72). Abdominal sterna IV-VI sparsely pubescent medially; apex of sternum VII more distinctly truncate in female than in male (Figs 105–106). Aedeagus slender, flattened (Fig. 130), lamella broadened to the left side, asymmetric (Fig. 129), thin and bent ventrally (Fig. 130).

#### Geographical distribution.

**Figure 158.**
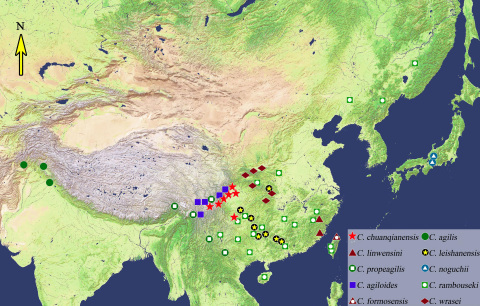
Map showing the known geographical distributions of *Chlaenius (Lithochlaenius)*spp.

Fig. 158. Confirmed only from Japan.

#### Remarks.

 Mensural data cited in the description were based on measurements obtained from 5 males and 5 females selected for maximum variation.

This species may well be endemic to Japan, and its reported occurrence in Korea (Kwon and Lee 1986) and Vietnam (Paik et al. 2006) requires further clarification. The slender aedeagus of males of this species is very different from the stout aedeagi of other species of the *agilis* group.

### Chlaenius (Lithochlaenius) wrasei

Kirschenhofer

http://species-id.net/wiki/Chlaenius_(Lithochlaenius)_wrasei

Figs 30–31 59 73 87 107–108 131–132 158

Chlaenius noguchii wrasei  Kirschenhofer, 1997:116, 118
Chlaenius wrasei  Kirschenhofer, 2005:491

#### Specimens examined.

 Total 41 specimens. **China:** 15 males and 6 females (IZCAS), "China, Shaanxi Prov., Zhouzhi, Houzhenzi, Shaliangzi, 33.88923˚N, 108.01553˚E”/ "907m, 2007.5.24, Shi Hongliang coll.”; 1 male and 1 female (IZCAS), "Shaanxi, Liuba, 1981.4”; 1 male and 1 female (IZCAS), "Shaanxi, Zhenba, 1981.4”; 1 female (IZCAS), "Shaanxi, Zhenba, 1985.7.19, Wang Shufang collector”; 1 male (IZCAS), "Shaanxi, Huaxian, 1980.5.4”; 1 male (IZCAS), "Shaanxi, Pingli, 1980.6.27”; 1 male and 1 female (IZCAS), "Shaanxi, Zhouzhi, Louguantai, light trap, 34.05378˚N, 108.29294˚E”/ "680m, 2008.6.22–26, Jiang Jianguo coll.”; 3 males and 5 females (HBUM), "Shaanxi, Gaolan County, Minzhu Township, 2003.7.4–5, Liu Yushuangand Yuan Caixia collectors; 1 male (IZCAS), "Hubei Prov. Xinshan, Xiakou, 140m”/ "1994.V.2, Li Wenzhu coll.”; 1 female (IZCAS), "Hubei Prov., Enshi, 437m”/ "light trap, 1980.V.26”; 1 female (IZCAS), "Ganshu Prov., Kangxian, Yangbalingchang, 1020m 1999.VII.10, Wang Hongjian coll.”

#### Diagnosis.

Antennomere 1 coniform (Fig. 59); antennomeres 1–3 brown; intervals 1–5 glabrous medially with one row of pubescence laterally near striae (Fig. 87); apical lamella of aedeagus moderately rounded (Fig. 131), thin and moderately reflexed in lateral view (Fig. 132).

#### Description.

Total length = 16.00–17.00 mm (mean = 16.4), width = 5.33–6.13 mm (mean = 5.68); HW = 2.70–2.95 mm (mean = 2.80), EYL = 1.00–1.05 mm (mean =1.01), ratio Ant3/Ant1 = 1.42–1.65 (mean = 1.52), PL/PW = 0.78–0.80 (mean = 0.79), EL/EW = 1.74–1.90 (mean = 1.80), EL/ PL= 1.37–1.47 (mean = 1.41).

Head and pronotum black with green and coppery metallic luster; elytra black; ventral surface black; antennomeres 4–11, trochanters, palpomeres, and tarsomeres dark brown; antennomeres 1–3, femora, tibiae yellow to brown.

Head with vertex sparsely pubescent and coarsely punctate behind eyes; antennomere 1 coniform (Fig. 59); labrum nearly truncate at apex. Pronotum with disk nearly smooth, very sparsely punctate near midline in a few specimens; basal foveae narrow, deep, sparsely punctate, pubescent. Elytra with intervals convex, glabrous medially, pubescent laterally (Fig. 87), intervals 6–9 densely pubescent; striae deep, punctate; humeral angle obtuse (Fig. 73). Abdominal sterna IV-VI nearly glabrous medially, sternum VII narrowly rounded at apex (107–108). Aedeagus stout (Fig. 132), lamella rounded at apex (Fig. 131), thin and slightly bent ventrally (Fig. 132).

#### Geographical distribution.

Fig. 158. Known from Shaanxi, Gansu, and Hubei Provinces, China.

#### Remarks.

 Mensural data cited in the description were based on measurements obtained from 5 males and 5 females selected for maximum variation.

*Chlaenius wrasei* was first described as a subspecies of *Chlaenius noguchii* by Kirschenhofer. Later (Kirschenhofer 2005), he upgraded this taxon to status as a distinct species. We agree with this decision, given that the pubescent elytral intervals 6–7 of adults and stout aedeagus of males are very different from those of *Chlaenius noguchii*.

Members of *Chlaenius wrasei* are similar to those of *Chlaenius leishanensis* in having antennomere 1 coniform and pronotum nearly impunctate, but differ from the latter in having intervals 1–5 wholly glabrous medially (only the basal portions of these intervals are glabrous medially in *Chlaenius leishanensis*), tibiae concolorous with femora (tibiae much darker than femora in *Chlaenius leishanensis*), and lamella of aedeagus thin and less bent ventrally (thickened and more markedly bent in *Chlaenius leishanensis*).

##### Therambousekigroup

### Chlaenius (Lithochlaenius) agiloides

Jedlička

http://species-id.net/wiki/Chlaenius_(Lithochlaenius)_agiloides

Figs 32–33 42 50 60 74 88 109–110 133–135 150 155 157–158

Chlaenius agiloides  Jedlička, 1935:5; Kirschenhofer, 1997:116; Kirschenhofer, 2005:490

#### Specimens examined.

 Total 603 specimens. **China:** 2 males (CCCC), "Yunnan, Weixi, Tacheng, light trap, 2006.8.22, Chen Jianren collector”; 2 females (IZCAS), "Sichuan, Wolong, 1900m, 1980.VIII.25, Liu Youjiao collector”; 1 male and 1 female (IZCAS), "Sichuan, Wolong, 1980.6.29, Bai Jiuwei collector”; 2 females (IZCAS), "Sichuan, Baoxing, 1400m”/ "1995.VIII.14, Yu Peiyu collector”; 4 males and 5 females (SIECAS), "Sichuan, Shimian, 2007.VII.20, alt. 900m, Liu, Zhang, Zhou & Bi”; 213 males and 204 females (IZCAS), "China, Sichuan Prov., Batang, Zhubalong, Sanjiacun, Jinshajiang, N29.84109, E99.02390”/2480m, 2009.6.1, Yuan Feng, Zhai Hui and Yang Ganyan collectors”; 71 males and 98 females (IZCAS), "China, Sichuan Prov.,, Yajiang, Hekou Town, Shanbeihou, Yalongjiang, N30.00020, E101.01526”/ "2583m, 2009.5.27, Liang Hongbin collector”.

#### Diagnosis.

Antennomere 1 cylindrical (Fig. 60); antennomeres 1–3 yellow; intervals punctate, pubescent (Fig. 88); lamella of aedeagus rounded at apex (Fig. 133), thick in lateral view (Figs 134–135).

#### Description.

Total length = 14.00–17.00 mm (mean = 15.10), width = 5.33–5.87 mm (mean = 5.60); HW = 2.40–2.85 mm (mean = 2.59), EYL = 0.95–1.00 mm (mean = 0.97), ratio Ant3/Ant1= 1.94–2.21 (mean = 2.08), PL/PW = 0.86–0.90 (mean = 0.88), EL/EW = 1.62–1.74 (mean = 1.68), EL/ PL = 1.30–1.39 (mean = 1.34).

Head, pronotum and elytra black, with blue or greenish blue metallic luster; ventral surface black; antennomeres 4–11, mandibles, palpomeres, trochanters, and tarsomeres brown; antennomeres 1–3, femora, and tibiae yellow.

Head convex, vertex punctate and pubescent behind eyes; antennomere 1 cylindrical (Fig. 60); labrum slightly emarginate or truncate at apex; mentum tooth emarginate apically. Pronotum with disk convex, sparsely punctate and pubescent medially, more densely punctate along midline, near lateral margins, and near base; basal foveae deep, punctate and pubescent. Elytra with intervals convex, wholly punctate and pubescent (Fig. 88), punctation and pubescence less dense in the basal half of intervals 1–4 than in other areas; striae moderately deep, punctate; humeral angle rounded or obtuse (Fig. 74). Abdominal sterna IV-VI sparsely pubescent medially; apex of sternum VII narrowly rounded in male (Fig. 109), slightly truncate in female (Fig. 110). Apical lamella of aedeagus moderately rounded (Fig. 133), thick in lateral view (Figs 134–135).

#### Geographical distribution.

Fig. 158. Known only from Sichuan and Yunnan Provinces, China.

#### Remarks.

 Mensural data cited in the description were based on measurements obtained from 5 males and 5 females selected for maximum variation.

Members of this species are very similar to *Chlaenius rambouseki* in having the elytra wholly pubescent, but differ from the latter in having shiny elytral intervals (dull in *Chlaenius rambouseki*), regular and uniform punctation on intervals (mixed large and small interval punctuation in*Chlaenius rambouseki*), and thick apical lamella of aedeagus in males (thin in *Chlaenius rambouseki* males). In many *Chlaenius agiloides* members, the dorsal surface has a blue metallic luster rather than the green luster of *Chlaenius rambouseki* members.

### Chlaenius (Lithochlaenius) rambouseki

Lutshnik

http://species-id.net/wiki/Chlaenius_(Lithochlaenius)_rambouseki

Figs 1–13 34–35 43–45 51 61, 65 75, 79 89, 93 111–112, 116 136–137 144–145, 151 152–154, 156 158

Chlaenius rambouseki  Lutshnik, 1933:172; Kirschenhofer, 2005:491
Chlaenius formosanus  Jedlička, 1935:5. **New synonymy**; Kirschenhofer, 1997:116; Kirschenhofer, 2005:490

#### Specimens examined.

 Total 66 specimens. **China:** 4 males and 5 females (IZCAS), "Heilongjiang, Hulin, Bank of Ussri River, 45.976578˚N, 133.669942˚E”/ "55m, 2009.5.20–24, night, Liu Ye collector”; 3 males and 8 females (OMNH), "Manchuria Fengtian (=Liaoning, Shenyang), 1942.V”/ "N. Tosawa collection, June 1978”; 1 female (HBUM), "Shaanxi, Langao County, Minzhu, 2003.7.4, Yuan Caixia and Liu Yushuang collectors”; 1 male (HBUM), "Henan, Songxian, Baiyunshan, 2008.7.14–17, Ren Guodong and Wu Qiqi collectors”; 1 female (IZCAS), "Fujian, Jiangyang, Huangkeng, Guilin, 270–340m”/ "1960.4.8, Ma Chenlin collector”; 1 male (CCCC), "China, Taiwan Prov., Hsinchu, Wufong, 1996.08.03, Chen C.C. coll.”; "1 male and 1 female (CCCC), "Taiwan Prov., Hsinchu, Wufong, 1998.4.11”; 3 males and 1 female (CCCC), "China, Taiwan Prov., Pingtung, Yitun, 2009.4.23”; 1 males and 1 female, "China, Taiwan, Pingtung, Yitun, 2008.6.29”, Chen C.C.”; 1 male (CCCC), "Taiwan, Pingtung, Manchou Harbor, 2008.10.30”; 1 female (CCCC), „China, Taiwan Prov., Yilan, 2007.6.10, Chen C.C.”; 1 male (CCCC), "China, Taiwan Privin., Chen C.C. collector”; 1 male (IZCAS), "Jiangxi, Liantang, light trap, 1956.8.8”; 1 male and 1 female (IZCAS), "Jiangxi, Liantang, 1956.6.10”; 1 male (IZCAS), "Jiangxi, Dayu, Lannijing, 550m, 1985.8.22, Liao Subai collector”; 1 male (IZCAS), "Jiangxi, Shangrao, 1980.8.7”; 1 male (IZCAS), "Hunan, Chansha, Yuelushan, 1955.7.15, Wang Linyao collector”; 2 males and 1 female (IZCAS), "Guangxi, Rongshui County, Yuanbaoshan, Tiantou village, 2009.10.26, Liu Ye and Shi Hongliang collectors”; 1 female (IZCAS), "Guangxi, Nanning, 1987.10.30, Zhou Zhihong collector”; 2 females (IZCAS), "Guangxi, Longsheng, Sanmen, 1983.4.8”; 1 male (HBUM), "Guangxi, Tian-e County, 2002.9.14–19, Bai Ming collector” 1 female (IZCAS), "Chongqing, Beibei, 1940.8.10”; 1 female (IZCAS), "China, Guizhou, Xishui County, Dabaitang, 600m”/ "2000.9.28, Liang H.B”; 1 male (HBUM), "Guizhou, Daozhen, Xiannvdong, Yang Xiujuan and Hua Huiran collectors”; 1 female (IZCAS), "Yunnan, Kunming, 1980.5”; 3 males and 3 females (IZCAS), "China, Hainan Prov., Baisha, Nankai, 19.08001˚N, 109.41058˚E”/ "259m, 2008.11.20 N, Shi H.L. collector”; 1 male and 4 females (IZCAS), "China, Hainan Prov., Baisha, Nankai River, bank, 19.08040˚N, 109.41267˚E”/ "255m, 2009.11.20 N, Liang Hongbin collector”. **Russia:**1 male and 1 female (ZRAS)**,**"Ussuri River, S of Bikin, Zvenjevaja [in Russian], 1982.7.28, Kabakov leg.”/ "*Chlaenius rambouseki* Lutshn. det. B. Kataev 2005”.**North Korea:**1 male and 1 female (IZCAS), Mt. Maedok Ridge, alt. 1538m Punso, Ryanggang province, North Korea”/ "NL40˚100, EL128˚20, 2007.7.1–20”.

#### Diagnosis.

Antennomere 1 elongate-ovoid (Figs 61, 65); antennomeres 1–3 more yellow; all intervals pubescent (Figs 89, 93); apical lamella of aedeagus moderately rounded (Figs 136, 144), thin in lateral view (Figs 137, 145).

#### Description.

Total length = 14.00–17.50 mm (mean = 15.60), width = 4.80–6.00 mm (mean = 5.60); HW = 2.40–2.95 mm (mean = 2.63), EYL = 0.95–1.00 mm (mean = 0.96), ratio Ant3/Ant1 = 1.96–2.10 (mean = 2.04), PL/PW = 0.86–0.90 (mean =0.87), EL/EW = 1.65–1.73 (mean = 1.68), EYL/ PL = 1.29–1.40 (mean = 1.35).

Head and pronotum black, with green or coppery metallic luster; elytra black in general, but with slight coppery luster in a few specimens; ventral surface black; antennomeres 4–11, mandibles, palpomeres, trochanters, and tarsomeres brown; antennomeres 1–3, femora and tibiae yellow.

Head convex, vertex punctate and pubescent behind eyes; antennomere 1 elongate–ovoid (Figs 61, 65); labrum slightly emarginate at apex; mentum with tooth emarginate apically. Pronotum with disk convex, sparsely punctate and pubescent along midline; basal foveae deep, coarsely punctate, pubescent. Elytra with intervals slightly convex, regularly punctate and pubescent (Figs 89, 93); striae shallow, punctate; humeral angle obtusely angulate (Figs 75, 79). Abdominal sterna densely pubescent; apex of sternum VII broadly rounded in both sexes (Figs 111–112, 116). Apical lamella of aedeagus rounded (Figs 136, 144), thickened in lateral view (Figs 154, 137, 145).

#### Geographical distribution.

Fig. 158. Broadly distributed across southern and central China, from Yunnan in the west to Hainan Island and Taiwan in the south, and northwest to North Korea and Primorski Krai in the Russian Far East.

#### Remarks.

 Mensural data cited in the description were based on measurements obtained from 5 males and 5 females selected for maximum variation.

We compared a photograph of the holotype (type lost according Kryzhanovskij 1976) and specimens of *Chlaenius formosanus* from Taiwan with identified specimens of *Chlaenius rambouseki* Lutshnik from Ussuri River, and no significant difference was found between them.

## Supplementary Material

XML Treatment for Lithochlaenius

XML Treatment for Chlaenius Lithochlaenius chuanqianensis

XML Treatment for Chlaenius (Lithochlaenius) linwensini

XML Treatment for Chlaenius (Lithochlaenius) propeagilis

XML Treatment for Chlaenius (Lithochlaenius) agilis

XML Treatment for Chlaenius (Lithochlaenius) formosensis

XML Treatment for Chlaenius (Lithochlaenius) leishanensis

XML Treatment for Chlaenius (Lithochlaenius) noguchii

XML Treatment for Chlaenius (Lithochlaenius) wrasei

XML Treatment for Chlaenius (Lithochlaenius) agiloides

XML Treatment for Chlaenius (Lithochlaenius) rambouseki
